# Development of Cassava Starch-Based Foams Incorporating Wastes from the Production of Organic Grape Juice and Grape Seed Oil

**DOI:** 10.1021/acsomega.4c10904

**Published:** 2025-02-18

**Authors:** Márcia Zanini, Wendel P. Silvestre, Camila Baldasso, Isabel C. Tessaro

**Affiliations:** 1 Graduate Program in Chemical Engineering (PPGEQ), Federal University of Rio Grande do Sul, Porto Alegre, RS 90040-060, Brazil; 2 Graduate Program in Process Engineering and Technologies (PGEPROTEC), University of Caxias do Sul, Caxias do Sul, RS 95070-560, Brazil

## Abstract

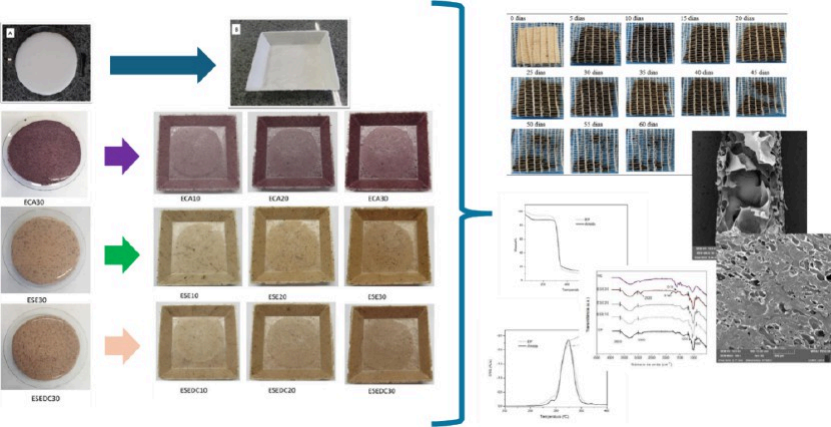

The generation of nonbiodegradable waste from food packaging is increasing. Due to the economic unfeasibility of waste segregation and recycling, this material ends up in landfills. To mitigate the environmental impact and explore alternative biodegradable materials, the present study seeks to develop foams based on cassava starch by incorporating agro-industrial residues from whole grape juice and organic grape seed oil for applications in food packaging. The starch-based foams were prepared by thermal expansion, using varying amounts of grape skin waste (CA), grape seed (SE), and defatted grape seed with grape skin fragments (SEDC) at proportions ranging from 10 - 30 wt %. The resulting foams were characterized concerning apparent density, moisture content, morphology, water absorption capacity, content of total phenolic compounds, total anthocyanins, total flavonoids, antioxidant capacity, thermogravimetric analysis (TGA), Fourier transform infrared spectroscopy (FTIR), and mechanical resistance via tensile testing. Incorporating CA, SE, and SEDC residues provided foams with lower apparent density and greater expansion capacity. Furthermore, the addition of SE and SEDC residues led to a lower water absorption capacity of the foams, while the addition of CA residue promoted an increased absorption capacity. Adding these residues increased the levels of total anthocyanins, total phenolic compounds, and flavonoids, resulting in higher antioxidant activity in the packaging compared to the control (without residues). Regarding thermal stability, the addition of CA, SE, and SEDC residue caused a decrease in the thermal stability of the foams compared to the control. FTIR spectra revealed the characteristic bands associated with the added residues. The tensile strength, elongation at break, and modulus of elasticity of the packaging with the addition of CA, SE, and SEDC residues were lower than the control sample and expanded polystyrene (EPS). Generally, the foams that included SE and SEDC residues performed better than those with CA residues. However, further studies are needed to optimize the formulation, mainly regarding water absorption capacity and mechanical resistance.

## 1 Introduction

Food packaging serves multiple functions essential for preserving food quality and safety. It acts as a barrier against factors responsible for chemical, physical, and microbiological deterioration during its shelf life.^1^ Additionally, it provides convenience for handling, transport, and storage.

Synthetic polymeric materials, such as polyethylene (PE), polypropylene (PP), polystyrene (PS), poly(vinyl chloride) (PVC), and poly(ethylene terephthalate) (PET), have been frequently used in food packaging. These materials are used for making films, flexible packaging, rigid containers, and foams.^2^ Expanded polystyrene (EPS) is particularly popular for food packaging foams due to its low cost, low density, high moisture resistance, and ease of use.^3^

However, the production of polymeric materials derived from petroleum has significant environmental impacts, such as the emission of greenhouse gases and pollution of soil and water. When it comes to disposal, these materials are often challenging to recycle, resulting in most being sent to landfills after a single use. This disposal causes environmental concerns, as discarded packaging comprises different materials with varying degradation times. It is estimated that only 4 % of discarded polymeric material is sent to landfills under controlled conditions.^4−6^

It is vital to search for biodegradable solutions and alternatives to replace plastic packaging made from nonbiodegradable sources, aiming to reduce the environmental impact caused by the disposal of nonbiodegradable polymeric waste in landfills.

Films composed of biomaterials are the most commonly used biodegradable packing materials. Their easy production and readily available feedstock make them interesting for such applications. However, their fragility and need for improved formulations are negative characteristics that hinder their widespread use. On the other hand, starch-based foams are reported as stable materials in dry environments, making them an interesting option for replacing synthetic packaging foams.^9−11^

Starch-based materials have great potential as biodegradable options for food packaging. The functional performance of starch-based biodegradable materials can be enhanced by adding other biopolymers, additives, or fillers and by employing innovative preparation techniques. However, economical large-scale production of high-performance starch-based biodegradable materials is still challenging, and further research is still needed.^7^

Starch also offers flexibility in foaming methods, making it possible to produce foam trays to replace single-use synthetic polymer packaging. Furthermore, there is growing interest in studying biodegradable materials made from starch and natural fibers for potential application.^3,8−10^ However, there are currently no reports on using grape pomace and grape seed residues individually in the formulation of cassava starch-based foams.

Several studies report the use of agricultural wastes in the production of biodegradable foams.^2,4,7,9,10^ However, the presence of bioactive compounds in the grape pomace/marc makes it an interesting and distinct kind of material, with the potential to provide functional compounds, increase the antimicrobial activity, or even reduce the degradation rates of the biopolymers and blends composed of this material.^10,11^

Although some studies address the use of grape wastes in producing biopolymer-based materials, most consider the waste a filler, considering it a mechanical reinforcement rather than a component that may provide distinct properties and characteristics to the material.^10,43,54^

This research aims to link food packaging with sustainability using low-environmental impact, biodegradable, renewable raw materials. It focuses on creating starch-based foams that incorporate waste from processing organic grapes. These formulations aim to create biodegradable packaging for food products by reusing waste from established production chains and utilizing renewable resources. This is one of the few studies that associated the use of a biopolymer and the waste from a productive chain with importance, especially in South Brazil, proposing a potential use for these materials.

## 2 Materials and Methods

### 2.1 Materials

The foams were prepared using sweet cassava starch (11 wt % moisture) sourced from Fritz and Frida, Ivoti, RS, Brazil. Cassava starch was chosen because cassava is widely available in Brazil and is a cheap starch source. The grape residues used were from the Ives variety (*Vitis labrusca* L.). They were of organic origin and were collected during the 2017-2018 harvest in March 2018 from Econatura Produtos Ecológicos e Naturais Ltda (Garibaldi, Brazil). The residues included skins (CA) and grape seeds (SE), as well as degreased seeds mixed with grape skin fragments (SEDC) from the organic grape seed oil manufacturing process. The characterization of these residues was previously detailed in a study by Zanini et al.^11^

The reagents used in the experiments, as well as their respective suppliers, were glycerol P.A. (Dinâmica, Brazil), guar gum P.A. (Labsynth, Brazil), and magnesium stearate P.A. (Dinâmica, Brazil). A metal mold measuring 140 mm × 140 mm × 2 mm was used to prepare the foams.^12^ This mold was coated with Teflon and included nine degassing outlets.

### 2.2 Methods

The foams were obtained using the thermocompression method. The formulations used to obtain the starch foams were adapted from Machado et al.,^3^ as compiled in Table 1.

**Table 1:** Formulations of Starch-Based Foams without Residue (Control) and Incorporating Different Residues in the Proportions of 10, 20, and 30 wt.%, Assessed in the Present Study

formulation	starch (wt %)^a^	water (wt %)^a^	residue (wt %)^a^	glycerol (wt %)^a^
EP	100	100	-	5
ECA10	90	119	10	10
ECA20	80	138	20	10
ECA30	70	157	30	10
ESE10	90	108.4	10	10
ESE20	80	116.8	20	10
ESE30	70	125.2	30	10
ESEDC10	90	112	10	10
ESEDC20	80	124	20	10
ESEDC30	70	136	30	10

a Relative to the total mass of solids. EP (standard starch foam), ECA10 (starch foam with 10 wt % grape skin), ECA20 (starch foam with 20 wt % grape skin), ECA30 (starch foam with 30 wt % grape skin), ESE10 (starch foam with 10 wt % grape seed), ESE20 (starch foam with 20 wt % grape seed), ESE30 (starch foam with 30 wt % seed grape), ESEDC10 (starch foam with 10 wt % defatted seed with skin fragments), ESEDC20 (starch foam with 20 wt % defatted seed with skin fragments), and ESEDC30 (starch foam with 30 wt % defatted seed with skin fragments).

In all formulations, 1.0 wt % of guar gum was added to prevent sedimentation of solids in the suspension, and 1.0 wt % of magnesium stearate as a release agent and antihumectant. The amount of water used in formulations with added residue was determined according to the water absorption capacity of each residue.

**Figure 1:**
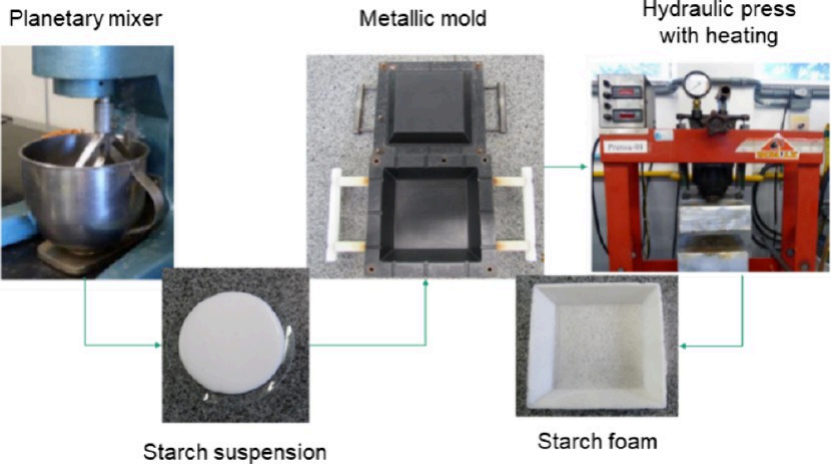
Steps of the process for obtaining the starch-based foams.

To obtain the foam samples, the materials in the formulations were weighed and mixed in a planetary mixer for 20 min. Afterward, 36 g of the resulting mass was placed into a metal mold and inserted into a hydraulic press under a constant pressure of 2.0 bar. The temperatures during this process ranged from 145 to 180 °C for 3–4 min. After completion, the produced foam was demolded. Figure 1 illustrates the main steps of the process for foam production.

The characterization tests for the obtained foams involved assessing several parameters, such as apparent density, moisture content, water absorption capacity, morphology, total phenolic compounds, anthocyanins, flavonoids, antioxidant activity, thermogravimetric analysis (TG), Fourier transform infrared spectroscopy (FTIR), and tensile strength.

The apparent density of the foams was determined according to the methodology established by Shogren et al.,^13^ which involves calculating the relationship between the mass (g) and the volume (cm^3^) occupied by the samples.

The moisture and volatile substance content of the starch-based foams was determined using the TG method, following the methodology described by the Adolfo Lutz Institute.^14^

Water absorption capacity was determined according to the Cobb methodology and aligned with the NBR/ISO 535 standard, with some adaptations.^15^ Samples measuring 25 mm × 50 mm were weighed and submerged in 100 mL of distilled water for different periods (1 min, 5 min, 10 min, 20 min, 30 min, and 60 min). After soaking, the samples were removed, and excess liquid was blotted off with absorbent paper. The wet samples were immediately weighed. The water absorption capacity was calculated based on the mass difference before and after soaking, and the results were expressed as a percentage of water mass absorbed per unit of dry sample mass.

The morphological characterization of the foams was performed by scanning electron microscopy (SEM) to analyze both the cross section and surface of the samples. This was performed with a scanning electron microscope (SSX-550, Shimadzu, Japan) at an acceleration voltage of 12 kV and magnifications of 50× and 100×. For sample preparation, the foams were cryogenically fractured using liquid nitrogen and then mounted on carbon tapes, and then a thin layer of gold was deposited.

The total phenolic compound content was determined using the Folin–Ciocalteau colorimetric method.^16,17^ The results were expressed in milligram equivalents of gallic acid (EAG) per 100 g of sample. The flavonoid content was determined by the aluminum chloride method, according to the procedure proposed by Matic et al., with results expressed in milligram equivalents of quercetin (EQ) per 100 g of sample.^18^ Total anthocyanin content was assessed using the differential pH method according to AOAC method 2005.02, and results were expressed in milligrams of cyanidin-3-glucoside equivalent per 100 g of sample.^19^

Antioxidant activity was evaluated by measuring the neutralization of the DPPH radical following the procedure described by Yamaguchi et al.^20^ The determination of antioxidant activity by neutralization of the ABTS^+^ radical followed the method proposed by Rufino et al.^21^ For these tests, a 5.0 mL aliquot of the hydroalcoholic extract used to determine phenolic compounds, flavonoids, and total anthocyanins was used as a sample.

TG tests were carried out on a thermogravimetric analyzer (TGA-50, Shimadzu, Japan) under an inert (N_2_) atmosphere with a flow rate of 50 mL·min^–1^. The heating rate was set at 10 °C·min^–1^, covering a temperature range from 25–900 °C, with approximately 10 mg of sample placed in a silicon carbide crucible. The differential thermogravimetry (DTG) curve was generated by the equipment software.

FTIR analyses were performed using a spectrophotometer (Nicolet iS10, Thermo Scientific, USA). Each spectrum was obtained with 32 scans, with wavenumbers ranging from 4000 - 400 cm^–1^ and a resolution of 0.25 cm^–1^, using the attenuated total reflection (ATR) method.

The tensile tests were conducted following the ASTM D 638-02a standard,^22^ with a claw separation of 50 mm and a tensile speed of 2.0 mm·s^–1^. The tests were carried out on a texturometer (TA.XT2i, Stable Micro Systems, United Kingdom) using a 50 N load cell. The test specimens had dimensions of 100 mm in length and 25 mm in width and were kept at 23 ± 2 °C and a relative humidity of 55 % forseven days before testing. From these tests, stress versus strain curves were obtained, allowing for the determination of rupture stress, elongation percentage, and modulus of elasticity.

Antimicrobial activity was assessed by determining microorganism survival. The drop plating assay was performed using the yeast *Candida albicans* CA01 and the bacteria *Escherichia coli* INCQS00033 and *Staphylococcus aureus* INCQS00015. The yeasts were maintained in YEPD medium (2.0 % w/v glucose, 1.0 % w/v yeast extract, 1.0 % w/v peptone, and 1.8 % w/v agar), while the bacteria were maintained in LB medium (1.0 % w/v tryptone, 0.5 % w/v yeast extract, 0.5 % w/v NaCl, and 1.8 % w/v agar).

The yeast was grown in liquid YEPD medium at 28 °C with orbital shaking until reaching the exponential growth phase (OD 600 nm ∼0.8) for the viability assays. Subsequently, the cells were washed with a saline solution (0.9 % w/v NaCl), and the cell concentration was adjusted to 1 × 10^7^ cells·mL^–1^. The bacteria were grown in a liquid LB medium at 28 °C under orbital shaking for 18 h. Then, the bacteria were washed with saline solution, achieving an optical density (OD) at 600 nm of 0.25.

The foam samples obtained were cut into squares measuring 1.0 cm × 1.0 cm and placed in Petri dishes containing agar-water medium. To each dish, 50 μL of the inoculum with previously adjusted cell concentration was added. The plates were then kept in humid chambers at 30 °C for 24 h. After this period, the fragments with the drops were transferred to tubes with 2 mL of 0.1% v/v Tween and vortexed for 30 s.

Viability assessments were performed by serial dilution (1:10) and plating per drop (5.0 μL) on YEPD plates for yeast and LB for bacteria. The plates were incubated at 28 °C for 24 h, and then the number of colony-forming units (CFUs) was counted. The results were expressed as a percentage of CFU compared to the control (EP).

The biodegradation test for the foams was conducted qualitatively, following the methodologies proposed by Medina Jaramillo et al.^23^ and Machado et al.,^3^ with adaptations. The foam samples, measuring 2.5 cm × 2.5 cm, were enclosed in a PP screen and buried to a depth of 40 mm in a PP box containing soil enriched with humus, kept at room temperature (20–25 °C). Water was sprayed daily to maintain soil moisture. Every 5 days, samples were removed from the soil for qualitative monitoring, which included visual inspection and photographic recording.

All analyses and experiments were performed in triplicate. Statistical analyses were conducted using the Statistical Package for the Social Sciences (SPSS), version 21.0 (IBM, USA). The results were classified as parametric, according to the Kolmogorov–Smirnov test. The results obtained were submitted to analysis of variance (ANOVA), followed by Tukey’s post hoc test at a 5 % probability of error (α = 0.05; *p* < 0.05).

## 3 Results and Discussion

**Figure 2:**
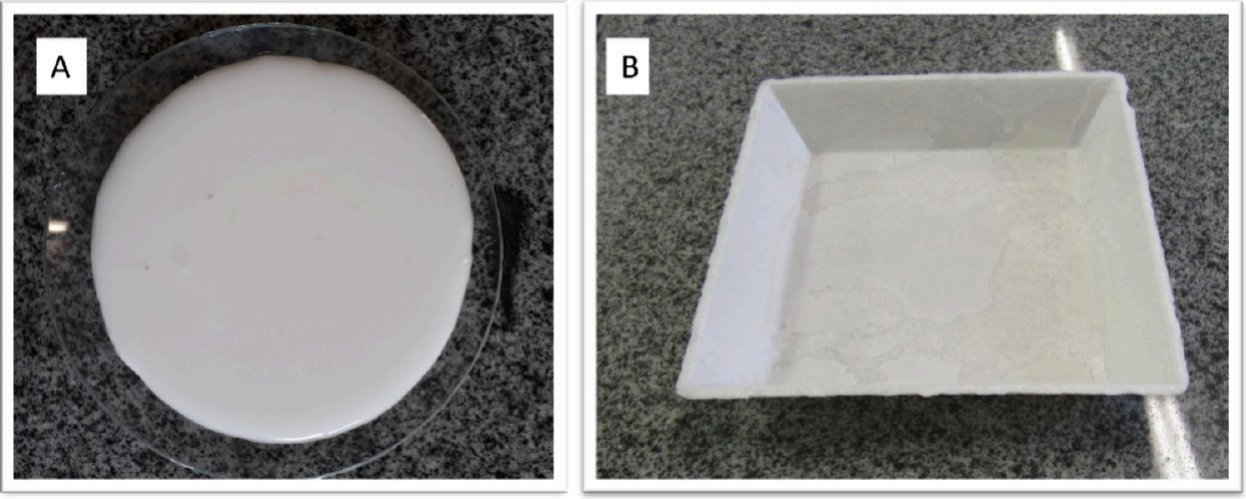
Images of the starch-based suspension (a) and the foam (b) obtained using the standard formulation.

For the standard formulation, the suspension and foam obtained can be seen in Figure 2. The suspension obtained was homogeneous, with no phase separation. Furthermore, the foam obtained was white after processing, with no cracks.

Regarding cassava starch-based foams that incorporate residues, the proposed methodology allowed for practical molding of the material by adding CA, SE, and SEDC residues. The percentage of these residues varied from 10 to 30 wt % relative to the total solids. However, it was not possible to incorporate higher percentages of waste due to problems in the demolding stage. At higher levels, the material became brittle and developed cracks, making it difficult to remove from the mold.

**Figure 3:**
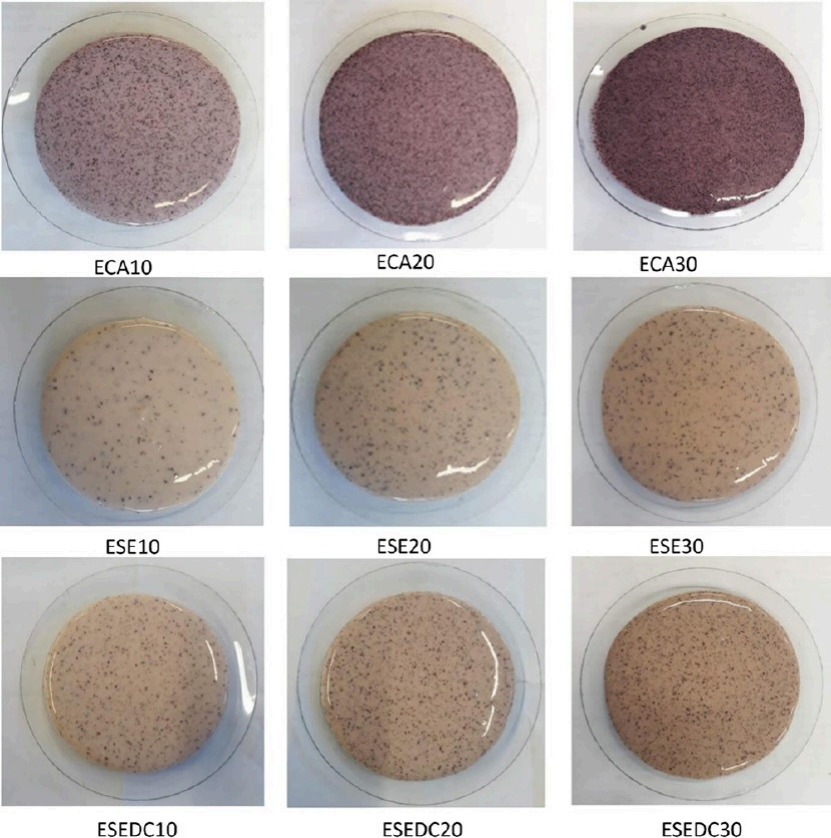
Images of suspensions based on cassava starch with different levels of incorporated residue, with the addition of grape skin residue: ECA10 (10 wt %), ECA20 (20 wt %), and ECA30 (30 wt %); with the addition of grape seed residue: ESE10 (10 wt %), ESE20 (20 wt %), and ESE30 (30 wt %); and with the addition of defatted seed residue with grape skin fragments: ESEDC10 (10 wt %), ESEDC20 (20 wt %), and ESEDC30 (30 wt %).

The starch suspensions with added residues had a homogeneous visual appearance, with no noticeable separation, as shown in Figure 3.

**Figure 4:**
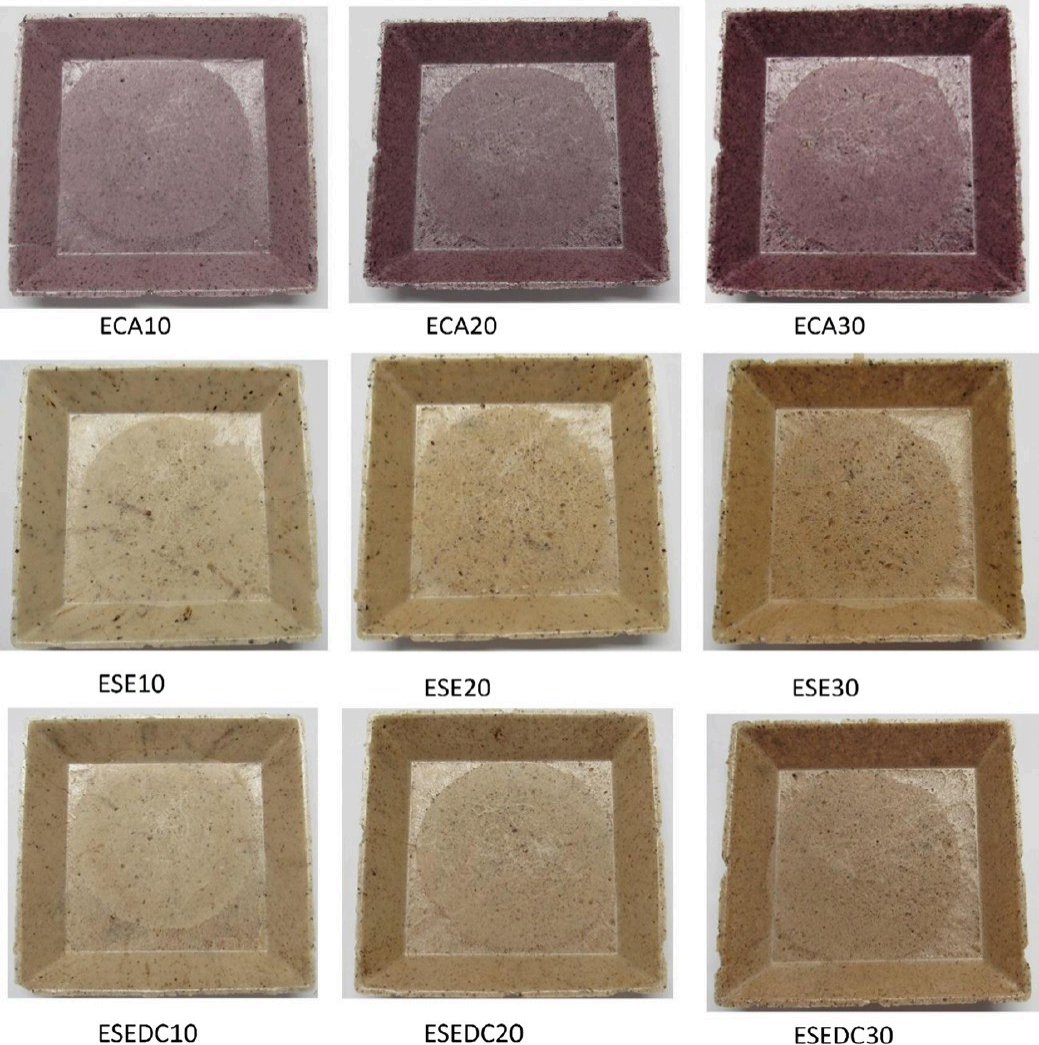
Images of foams based on cassava starch and incorporated with different residue levels obtained, EP (standard starch foam), with the addition of grape skin residue: ECA10 (10 wt %), ECA20 (20 wt %), ECA30 (30 wt %); with the addition of grape seed residue: ESE10 (10 wt %), ESE20 (20 wt %), ESE30 (30 wt %), and with the addition of defatted seed residue with grape skin fragments: ESEDC10 (10 wt %), ESEDC20 (20 wt %), and ESEDC30 (30 wt %).

The foams exhibited the characteristic color of the added residue, which became more intense with an increase in the percentage of incorporated residue. The foams can be seen in Figure 4.

It is important to ensure that the amount of suspension added to the mold is adequate. Excessive amounts may lead to an increase in steam release, resulting in fragile and brittle trays.^24^

For starch suspensions containing 30 wt % residues, it was necessary to reduce the temperature to 145 °C and reduce the residence time in the mold to 3 min to prevent the foam from breaking during the demolding process. It was observed that the greater the percentage of residue incorporated, the greater the color intensity in the foam. Furthermore, the color did not change with heat application, indicating that the processing temperature likely did not harm the waste’s properties.

### 3.1 Bulk Density and Moisture Content

The results regarding the apparent density and moisture content of foams developed based on cassava starch, incorporating varying levels of CA, SE, and SEDC residues, are summarized in Table 2.

**Table 2:** Bulk Density and Moisture Content of Foams Based on Cassava Starch and Incorporated with Different Residue Levels: EP (Standard Starch Foam), with the Addition of Grape Skin Residue: ECA10 (10 wt.%), ACE20 (20 wt.%), ACE30 (30 wt.%); with the Addition of Grape Seed Residue: ESE10 (10 wt.%), ESE20 (20 wt.%), ESE30 (30 wt.%); and with the Addition of Defatted Seed Residue with Grape Skin Fragments: ESEDC10 (10 wt.%), ESEDC20 (20 wt.%), and ESEDC30 (30 wt.%)^a^

formulation	bulk density (g·cm^–3^)	moisture content (wt.%)
EP	0.205 ± 0.005a	2.30 ± 0.33d
ECA10	0.143 ± 0.002d	4.88 ± 0.52c
ECA20	0.141 ± 0.001d	6.70 ± 0.26b
ECA30	0.118 ± 0.003e	8.14 ± 0.46a
ESE10	0.176 ± 0.003b	5.61 ± 0.18c
ESE20	0.164 ± 0.002c	6.58 ± 0.24b
ESE30	0.147 ± 0.002d	7.42 ± 0.22a
ESEDC10	0.171 ± 0.003b	6.24 ± 0.17b
ESEDC20	0.163 ± 0.010c	6.74 ± 0.13b
ESEDC30	0.146 ± 0.005d	7.73 ± 0.51a

a Means in the same column with different lowercase letters show significant differences using the Tukey test at a 5% probability of error (*p* < 0.05).

The bulk density of the foams ranged from 0.118 - 0.205 g·cm^–3^. In all samples, adding residues decreased apparent density, with a significant difference observed between the residue-added samples and the control sample (EP).

This outcome can be attributed to the residues acting as reinforcing fillers, providing greater expansion capacity of the starch suspensions and resulting in more expandable materials. When starch is processed without additives or reinforcements, stiffer materials are produced, as this biopolymer does not support cell growth during foam formation.^24^

Those with CA residue exhibited the lowest apparent density values among the foams. Such a behavior can be explained by the higher water absorption capacity of CA residue, leading to suspensions with higher water contents when compared to those containing SE and SEDC residues. ECA30 foam achieved the lowest apparent density value among all formulations. For ECA10 and ECA20 foams, no significant difference was observed between them.

Compared to the formulations containing the SEDC and SE residues, the foams ESEDC30 and ESE30 exhibited the lowest bulk densities, measuring 0.146 - 0.147 g·cm^–3^, respectively, with no significant difference between the samples. A similar trend was observed with the addition of SEDC and SE, as there was no significant difference in bulk density when analyzing incorporation percentages of 10, 20, and 30 wt.%. Most probably, the expansion was directly attributed to the water content of the residue, even after drying, as observed in Table 2.

Vercelheze et al.^24^ reported that adding sugarcane fiber and nanoclays to cassava starch foams decreased the bulk density, ranging from 0.194 - 0.296 g·cm^–3^. Machado et al.^3^ reported bulk density values ranging from 0.23 - 0.30 g·cm^–3^, evaluating cassava starch foams with the addition of sesame seed cake.

The foams developed in this study had higher bulk density values than commercial PS foams, which typically have a density ranging between 0.04 and 0.06 g·cm^–3^.^3,24^ However, the addition of residues resulted in a reduction of 14 % to 42% in bulk density compared to the control sample (EP).

Cabanillas et al.^9^ reported bulk density values ranging from 0.368 - 0.410 g·cm^–3^ for starch foams adding 5 wt.% pineapple peel fibers, noting that the incorporation of fiber did not significantly affect the apparent density compared to the control sample.

Regarding moisture content, adding residues to the foams increased moisture content by 112 – 267 % compared to the control sample (EP). Such behavior may be attributed to reducing the thermopressing time during processing to prevent the foams from rupturing during demolding.

The foams containing 30 wt.% waste (ECA30, ESE30, and ESEDC30) exhibited the highest moisture content, with no significant difference. The addition of waste at this level resulted in the most prominent reduction in thermoforming time, although the foams produced were more challenging to demold.

### 3.2 Morphology

**Figure 5:**
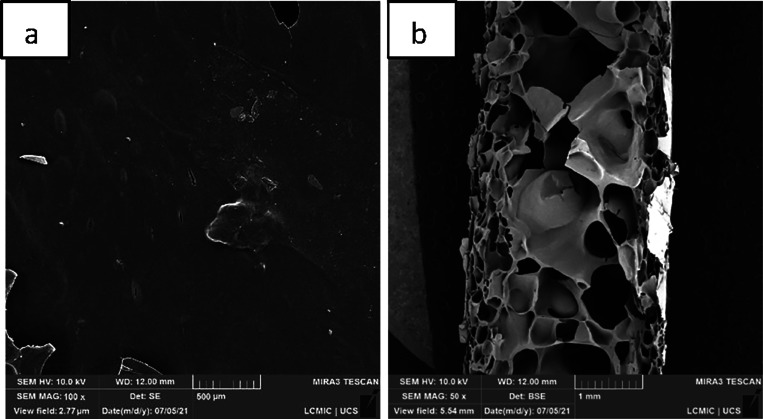
Micrographs, obtained by SEM, of cassava starch foam, standard sample (EP): (a) surface, with 100× magnification; (b) cross section, magnified 50×.

**Figure 6:**
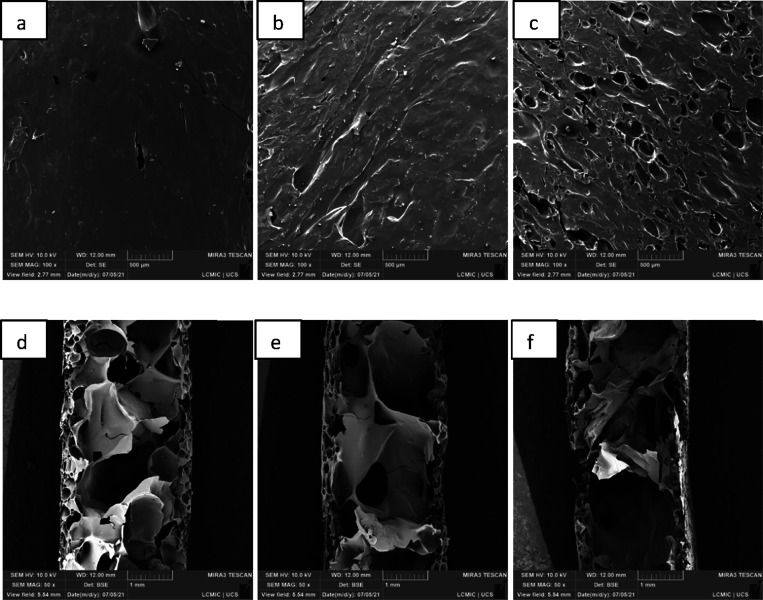
Micrographs, obtained by SEM, of the cassava starch foam with the incorporation of grape skin residue (CA) from the surface, with 100× magnification: (a) ECA10 (10 wt.%); (b) ECA20 (20 wt.%); and (c) ECA30 (30 wt.%), and cross section at 50× magnification: (d) ECA10 (10 wt.%); (e) ECA20 (20 wt.%); (f) ECA30 (30 wt.%).

**Figure 7:**
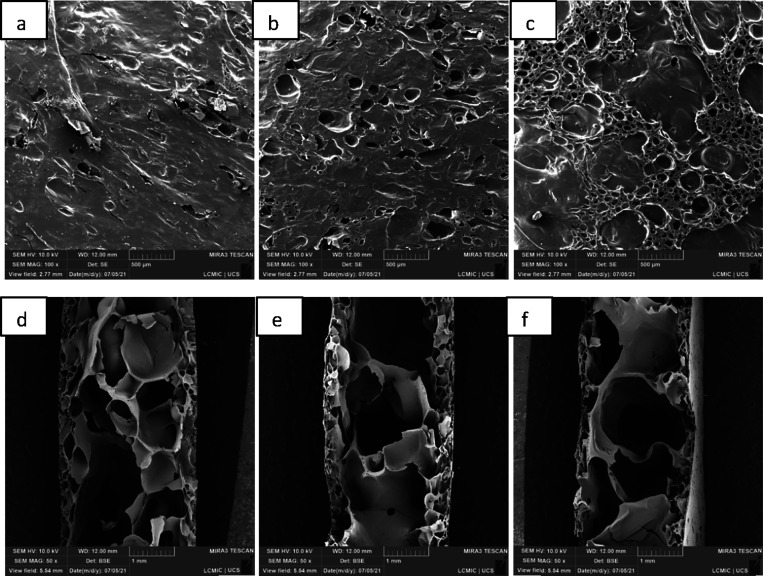
Micrographs, obtained by SEM, of the cassava starch foam with the incorporation of the grape seed residue (SE) on the surface, with 100× magnification: (a) ESE10 (10 wt.%); (b) ESE20 (20 wt.%); and (c) ESE30 (30 wt.%) and cross-section at 50× magnification: (d) ESE10 (10 wt.%); (e) ESE20 (20 wt.%); (f) ESE30 (30 wt.%).

**Figure 8:**
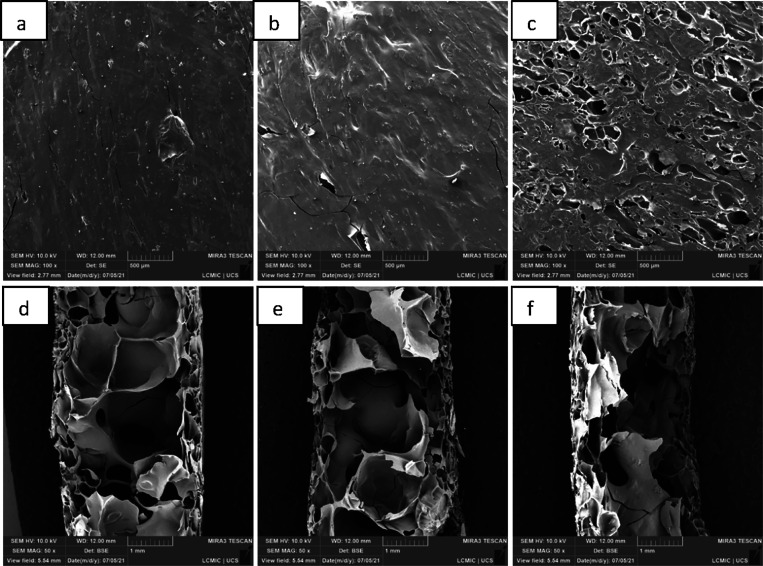
Micrographs, obtained by SEM, of the cassava starch foam with the incorporation of the defatted grape seed residue with grape skin fragments (SEDC) from the surface, with 100× magnification: (a) ESEDC10 (10 wt.%); (b) ESEDC20 (20 wt.%); and (c) ESEDC30 (30 wt.%), and cross section at 50× magnification: (d) ESEDC10 (10 wt.%); (e) ESEDC20 (20 wt.%); (f) ESEDC30 (30 wt.%).

The microstructures of the cross section and surface of the cassava starch foams, both from the standard sample (EP) and those with the addition of CA, SEDC, and SE residues, are shown in Figures 5, 6, 7, and 8, with magnifications of 50× and 100×.

In the micrographs of the cross sections from all samples, a cellular structure featuring two distinct layers is observed: an outer layer and an inner layer. The outermost layers have a denser structure, characterized by smaller cell sizes and more empty spaces, while the inner layer has larger cells and a more expanded structure. According to Jarpa-Parra and Chen, these structures are typical for foams produced by mold compression.^25^

The proximity to the hot mold resulted in foams with a denser outer layer and a smaller cellular structure. In contrast, the internal area has a larger cellular structure due to the amount of water inside the mold in the form of steam, promoting the expansion and rupture of the cells.^3^

Micrographs revealed that incorporating residues from CA, SE, and SEDC modified the microstructure of the standard foam. In the cross-section analysis, the residues provided more compact external layers and larger voids in the internal areas. It is also observed that a higher percentage of incorporated residue led to greater compaction near the surface and an increase in the number of empty spaces within the middle of the structure. These differences may be related to the greater amount of water in the suspensions and the reinforcing properties of the waste, increasing the void spaces’ size. Similar findings were reported in studies by Vercelheze et al.^24^ and Engel et al.^10^

Surface micrographs indicated that foams containing 10 wt.% and 20 wt.% CA and SEDC residues presented a more compact surface than the control sample (EP). On the other hand, foams with the addition of 30 wt.% SEDC and SE residues showed more discontinuous and irregular regions. This irregularity may be associated with the presence of grape seed oil in the first layers of the foam due to the formation of starch–lipid complexes during thermal processing.

Cruz-Tirado et al.^26^ reported that starch and oil interact chemically during gelatinization, encapsulating the oil within the starch structure. Subsequently, steam displaces the oil to the outermost layers of the foam. Due to the hydrophobic nature of the oil, water vapor trapped within the foam causes deformation but not cracking.

Additionally, Cruz-Tirado et al. noted that small oil droplets may be trapped inside the starch granules, forming oil-starch complexes in the foam layers.^26^ These oil droplets probably interact with the starch bonds, becoming incorporated into the polymeric matrix within the foam structure, mainly in the initial thick layer formed during drying. As a result, they can provide an antimicrobial effect on the foam.

### 3.3 Water Absorption Capacity

The results concerning the water absorption capacity of foams developed using cassava starch, based on the standard formulation and different levels of CA, SE, and SEDC residues, are presented in Table 3.

**Table 3:** Water Absorption Capacity (wt.% on a Dry Basis) Depending on the Exposure Time of Foams Based on Cassava Starch and Incorporated with Different Residue Levels: EP (Standard Starch Foam), with Addition from Grape Skin Residue: ECA10 (10 wt.%), ECA20 (20 wt.%), and ECA30 (30 wt.%); with the Addition of Grape Seed Residue: ESE10 (10 wt.%), ESE20 (20 wt.%), and ESE30 (30 wt.%); and with the Addition of Defatted Seed Residue with Grape Skin Fragments: ESEDC10 (10 wt.%), ESEDC20 (20 wt.%), and ESEDC30 (30 wt.%)^a^

sample	1 min	5 min	10 min	20 min	30 min	60 min
EP	23.5 ± 2.5b	58.2 ± 1.4c	77.5 ± 3.1c	111.4 ± 3.0c	174.8 ± 3.8c	290.9 ± 4.8c
ECA10	52.4 ± 2.9a	68.2 ± 2.4b	103.1 ± 3.2b	123.3 ± 2.9b	187.1 ± 2.1b	306.7 ± 5.7b
ECA20	53.1 ± 1.7a	88.9 ± 2.6a	114.3 ± 5.3a	133.4 ± 5.2a	215.9 ± 5.4a	324.1 ± 3.6a
ECA30	54.2 ± 2.5a	92.9 ± 1.7a	118.7 ± 2.8a	139.6 ± 4.0a	220.6 ± 3.8a	329.1 ± 2.3a
ESE10	24.1 ± 1.3b	49.3 ± 2.7d	63.0 ± 3.7d	76.2 ± 2.7e	100.4 ± 2.4d	121.7 ± 2.9f
ESE20	23.7 ± 2.1b	48.0 ± 2.9d	62.0 ± 3.8d	74.6 ± 1.2e	98.0 ± 3.2d	119.4 ± 3.7f
ESE30	20.6 ± 2.3b	39.1 ± 2.6e	56.1 ± 1.0e	70.8 ± 1.8f	91.1 ± 2.8e	107.1 ± 3.7g
ESEDC10	25.8 ± 3.1b	53.1 ± 1.2d	65.4 ± 1.4d	90.5 ± 4.3d	105.9 ± 4.5d	137.5 ± 1.6d
ESEDC20	25.3 ± 2.7b	50.1 ± 2.5d	64.7 ± 3.5d	84.1 ± 3.3d	103.2 ± 4.3d	134.4 ± 2.5d
ESEDC30	24.9 ± 2.2b	47.8 ± 2.3d	62.3 ± 0.5d	83.2 ± 3.8d	99.2 ± 2.1d	126.1 ± 1.7e

a Means in the same column with different lowercase letters show significant differences using the Tukey test at a 5% probability of error (*p* < 0.05).

The addition of CA residue in forming starch foams resulted in a greater water absorption capacity at concentrations of 10, 20, and 30 wt.%. There was a significant difference between the EP and ECA10, ECA20, and ECA30 samples. Furthermore, for the concentration of 30 wt.% CA, the water absorption capacity varied in the 13 – 130 % range compared to the EP sample, with the most remarkable difference observed at 1 min and the smallest at 60 min.

Regarding the variation in the water absorption capacity of the foams and the increase in the percentage of CA residue for 1 min, no significant difference was noted among the samples. The ECA10 sample showed a significant difference in water absorption capacity at other evaluated time intervals compared to the ECA20 and ECA30 samples. For the ECA20 and ECA30 samples, there was no significant difference in water absorption capacity relative to exposure time.

The enhanced water absorption capacity in foams containing CA residue can be attributed to the lower apparent density of the foams, allowing greater water absorption in shorter immersion times. Additionally, the chemical composition of this residue, as it has lower insoluble fiber content (cellulose and lignin) and lipids compared to SE and SEDC residues, aligns with the increased water absorption capacity, as previously reported.^11^

In contrast, adding SE residue in forming starch foams provided a lower water absorption capacity at concentrations of 10, 20, and 30 wt.%. A significant difference was found between the EP sample and the ESE10, ESE20, and ESE30 samples, except for the 1 min measurement. Furthermore, the biggest differences in water absorption capacity compared to the EP sample were observed at the 30 wt.% SE concentration, with values ranging from 33 - 63 %. The smallest difference was measured at 5 min, while the largest was recorded at 60 min.

Considering the variation in water absorption capacity among the foams and the increased percentage of SE residue at 1 min, there was no significant difference between the samples. For the ESE10 and ESE20 samples, variations in immersion time did not lead to significant differences in water absorption capacity. However, the ESE30 sample presented the lowest water absorption capacity among all analyzed samples, presenting a significant difference from the other samples, except at the 1 min measurement.

Adding SEDC residue into the starch foams resulted in lower water absorption capacity at concentrations of 10, 20, and 30 wt.%. There was a significant difference between the EP and ESE10, ESE20, and ESE30 samples, except for the 1 min measurement. Furthermore, the differences in the EP sample compared to the 30 wt.% SEDC concentration, the percentage values for water absorption capacity were lower, ranging from 18 - 55 %. The smallest difference occurred within the first 5 min, while the largest difference was at 60 min.

Additionally, when comparing the EP sample with the 30 wt.% SEDC concentration, the percentage values were lower, ranging from 18 - 55 %. The slightest difference was the first 5 min, 60 min mark.

Regarding the variation in the water absorption capacity of the foams and the increase in the percentage of SEDC residue, no significant differences were observed between the ESEDC10, ESEDC20, and ESEDC30 samples, except for the ESEDC30 sample at the time of 60 min.

The lower water absorption capacity in foams with added SE and SEDC residues may be related to their higher lipid content,^11^ providing an increase in the hydrophobicity of the foam and contributing to a reorganized starch structure with fewer exposed hydroxyl groups. Furthermore, the presence of fibers might have further reduced the water absorption capacity of starch-based foams. This can be explained by the chemical nature of cellulose, which is poorly soluble in water.^24,27^

Machado et al.^3^ reported a decrease in the water absorption capacity of cassava starch foams with the addition of sesame seed cake, probably due to the higher lipid and protein content in the cake incorporated into the biopolymer.

Similarly, Berger et al. found that high-density polyethylene (HDPE) compounds containing grape pomace residue exhibited lower moisture absorption.^28^ The authors attributed these findings to the chemical composition of the grape biomass, specifically the presence of hydrophobic phenols and the oil from the seeds.

### 3.4 Contents of Total Phenolic Compounds, Anthocyanins, and Flavonoids

Table 4 displays the results concerning the contents of total phenolic compounds, total anthocyanins, and flavonoids in the foams.

**Table 4:** Contents of Total Phenolic Compounds, Total Anthocyanins, and Flavonoids of Foams Based on Cassava Starch and Incorporated with Different Residue Levels: EP (Standard Starch Foam), with the Addition of Grape Skin Residue: ECA10 (10 wt.%), ECA20 (20 wt.%), and ECA30 (30 wt.%); with the Addition of Grape Seed Residue: ESE10 (10 wt.%), ESE20 (20 wt.%), and ESE30 (30 wt.%); and with the Addition of the Defatted Seed Residue with Grape Skin Fragments: ESEDC10 (10 wt.%), ESEDC20 (20 wt.%), and ESEDC30 (30 wt.%)^a^

sample	anthocyanins (mg_EC_·100 g^–1^)	phenolic compounds (mg_EAG_·100 g^–1^)	flavonoids (mg_EQ_·100 g^–1^)
EP	<0.1 ± 0.00h	<0.10 ± 0.00i	4.49 ± 1.86g
ECA10	9.53 ± 0.20b	48.38 ± 1.41f	29.64 ± 1.46f
ECA20	14.11 ± 1.51a	64.41 ± 2.12e	33.46 ± 1.15f
ECA30	14.23 ± 0.20a	119.41 ± 2.77b	41.48 ± 2.07e
ESE10	4.45 ± 0.09f	34.97 ± 0.26h	30.16 ± 1.16f
ESE20	6.30 ± 0.12e	69.19 ± 0.51d	59.76 ± 1.95d
ESE30	8.76 ± 0.36c	93.23 ± 2.65c	72.79 ± 3.55c
ESEDC10	2.06 ± 0.26g	39.87 ± 1.06g	34.33 ± 3.94f
ESEDC20	4.52 ± 0.49f	116.13 ± 3.60b	83.19 ± 2.85b
ESEDC30	7.33 ± 0.20d	159.27 ± 1.32a	116.54 ± 1.98a

a EC—cyanidin-3-glucoside equivalents; EAG—gallic acid equivalents; EQ—quercetin equivalents. Means in the same column with different lowercase letters show significant differences using the Tukey test at a 5 % probability of error (*p* < 0.05).

Adding CA, SE, and SEDC residues to the starch foams provided greater amounts of total anthocyanins, phenolic compounds, and flavonoids, with a significant difference compared to the control sample (EP).

Considering the total anthocyanin content, samples ECA20 and ECA30 had the highest levels, 14.11 mg_EC_·100 g^–1^ and 14.23 mg_EC_·100 g^–1^, respectively, with no significant difference between the samples. The result demonstrates that the CA residue has the highest percentage of total anthocyanins.^11^ Notably, despite the high temperature during thermocompression, the anthocyanins, and thus the color, were effectively preserved in the resulting foams.

The ESEDC30 sample presented the highest concentration of total phenolic compounds and flavonoids, corresponding to levels of 159 mg _EAG_·100 g^–1^ and 116.5 mg _EAG_·100 g^–1^, respectively, with a significant difference from the other samples. This result confirms that the SEDC residue has the highest total phenolic compounds and flavonoid levels among the residues analyzed.^11^ Additionally, concerning flavonoids, the samples containing 10 wt % CA, SE, and SEDC residues did not differ significantly.

As the percentage of added materials increased, the quantified levels of total anthocyanins, phenolic compounds, and flavonoids also increased. Furthermore, for samples with added SE and SEDC residues, significant differences were noted across all added percentages.

As reported by Berger et al., anthocyanins can impart valuable properties to composites, including thermal stability, antioxidant capacity, and biocidal characteristics.^28^ These properties help reduce degradation caused by microorganisms and UV radiation, extending the useful life of polymer composites and their blends.

### 3.5 Determination of Antioxidant Activity

The results of the antioxidant activity of the foam samples based on the percentage of DPPH and ABTS^+^ free radicals scavenging, as well as the respective equivalent levels in millimolars of Trolox, are shown in Table 5.

**Table 5:** Antioxidant Capacity of Foams Based on Cassava Starch and Incorporated with Different Residue Levels: EP (Standard Starch Foam), with the Addition of Grape Skin Residue: ECA10 (10 wt.%), ECA20 (20 wt.%), and ECA30 (30 wt.%); with the Addition of Grape Seed Residue: ESE10 (10 wt.%), ESE20 (20 wt.%), and ESE30 (30 wt.%); and with the Addition of Defatted Seed Residue with Grape Skin Fragments: ESEDC10 (10 wt.%), ESEDC20 (20 wt.%), and ESEDC30 (30 wt.%)^a^

sample	DPPH radical sweeping (%)	mM Trolox equivalent (DPPH)	ABTS radical sweeping (%)	mM Trolox equivalent (ABTS)
EP	7.47 ± 1.71g	0.10 ± 0.03	12.97 ± 0.8f	0.18 ± 0.006
ECA10	9.25 ± 1.77f	0.13 ± 0.01	30.84 ± 0.08e	0.48 ± 0.025
ECA20	10.33 ± 0.10f	0.15 ± 0.01	33.41 ± 0.53e	0.51 ± 0.019
ECA30	16.34 ± 0.98e	0.22 ± 0.03	38.16 ± 1.36d	0.58 ± 0.018
ESE10	8.46 ± 3.35f	0.15 ± 0.07	36.50 ± 0.60d	0.54 ± 0.014
ESE20	33.27 ± 1.18d	0.49 ± 0.01	49.47 ± 2.71c	0.75 ± 0.028
ESE30	47.33 ± 0.30c	0.53 ± 0.01	53.92 ± 0.38b	0.83 ± 0.020
ESEDC10	31.76 ± 0.71d	0.47 ± 0.01	31.88 ± 1.00e	0.48 ± 0.018
ESEDC20	53.35 ± 1.22b	0.79 ± 0.03	49.10 ± 0.38c	0.73 ± 0.016
ESEDC30	66.77 ± 1.37a	0.98 ± 0.05	67.72 ± 2.41a	1.03 ± 0.015

a Different letters in the same column indicate significant differences between means using the Tukey test at a 5 % probability of error (*p* < 0.05).

Adding CA, SE, and SEDC residues to the foams significantly enhanced their antioxidant capacity, as demonstrated by the scavenging of DPPH and ABTS^+^ free radicals, compared to the control sample (EP).

Using the DPPH method, scanning percentages ranged from 7.5 % (EP) to 66.8 % (ESEDC30). Samples ECA10, ECA20, and ESE10 did not show significant differences in the neutralization of the DPPH radical, like the samples ESEDC10 and ESE20. The ESEDC30 sample presented the highest antioxidant capacity, with a sweep percentage of 66.8 %.

As observed in Table 5, higher percentages of residue added resulted in greater antioxidant capacity for all samples. When analyzing each residue individually, the samples with 30 wt % of the residue yielded the highest antioxidant capacity values, showing a significant difference compared to other samples. Scanned by the DPPH method, they were 2.2 (ECA30), 4.7 (ESE30), and 8.9 (ESEDC30) times greater than the standard sample (EP), respectively.

For the ABTS^+^ radical method, the scavenging percentages ranged from 13.0 - 67.7 %. Samples ECA10, ECA20, and ESEDC10 did not show a significant difference, nor did samples ECA30 and ESE10 and ESE20 and ESEDC20. The sample ESEDC30 again showed the highest antioxidant capacity (67.7 %). For samples with the addition of 30 wt % of residue, the antioxidant activity values increased by 2.9 times (ECA30), 4.2 times (ESE30), and 5.2 times (ESEDC30) relative to the control sample (EP).

Using both methods, foams made with SEDC residue showed higher antioxidant capacity values than those made with CA and SE residues. Notably, SEDC residue contained higher levels of flavonoids and total phenolic compounds, which are responsible for the antioxidant activity of grape residues.^11^

The potential use of grape processing residue as a natural antioxidant in food packaging has been highlighted by Cejudo-Bastante et al.,^29^ who used grape pomace extract (Petit Verdot, a *Vitis vinifera* variety) as a natural antioxidant in the development of bioactive jute fibers for food packaging, obtaining a more efficient material in terms of food preservation.

Furthermore, Gasiński et al.^30^ and Nakov et al.^31^ reported that adding grape pomace to beer and cake production increased the antioxidant capacity of the final products, bringing benefits in terms of nutraceutical potential and product preservation.

### 3.6 Thermogravimetric analysis

TG analysis was conducted to determine the effect of waste addition on the thermal stability of the foams. The results were analyzed based on the mass losses of the foams observed in three temperature ranges: 150–310 °C, 310–400 °C, and the residual mass at 900 °C.

**Figure 9:**
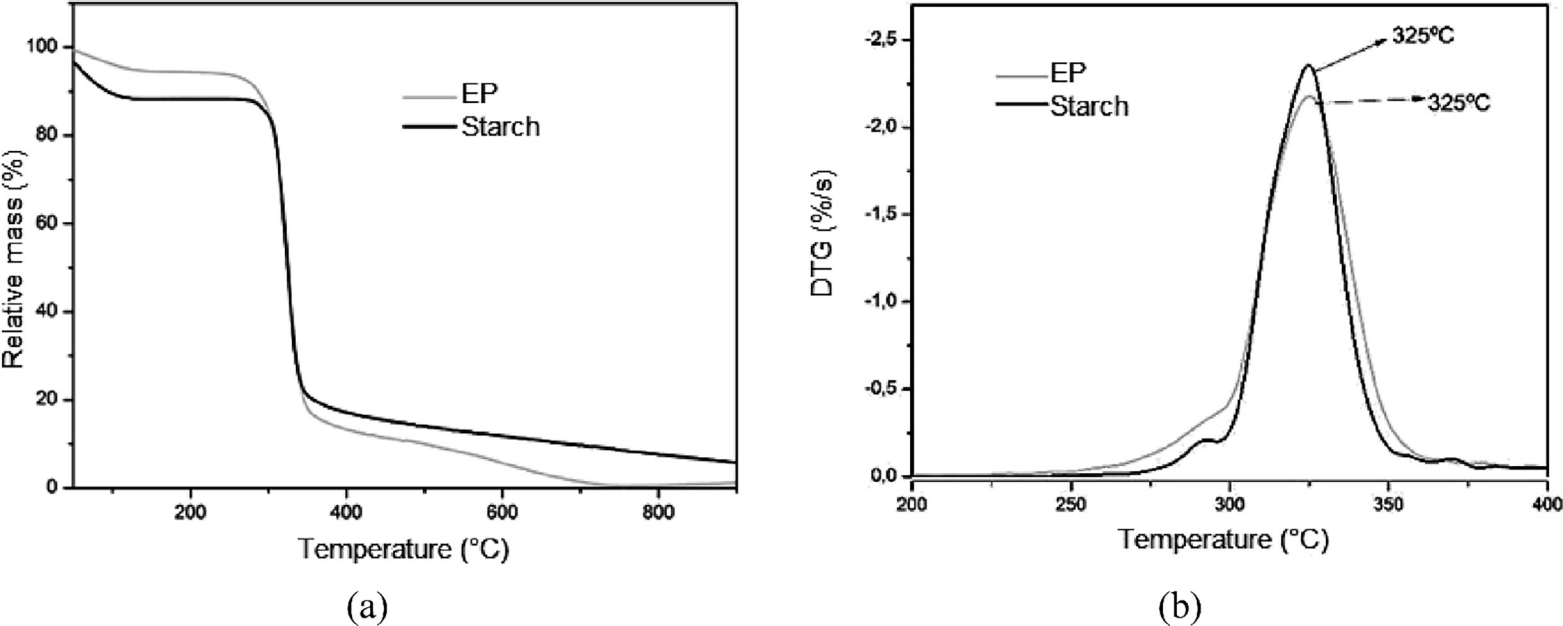
Thermogravimetric analysis (a) and first derivative of the mass loss (b) of standard starch foam (EP), compared to cassava starch.

**Figure 10:**
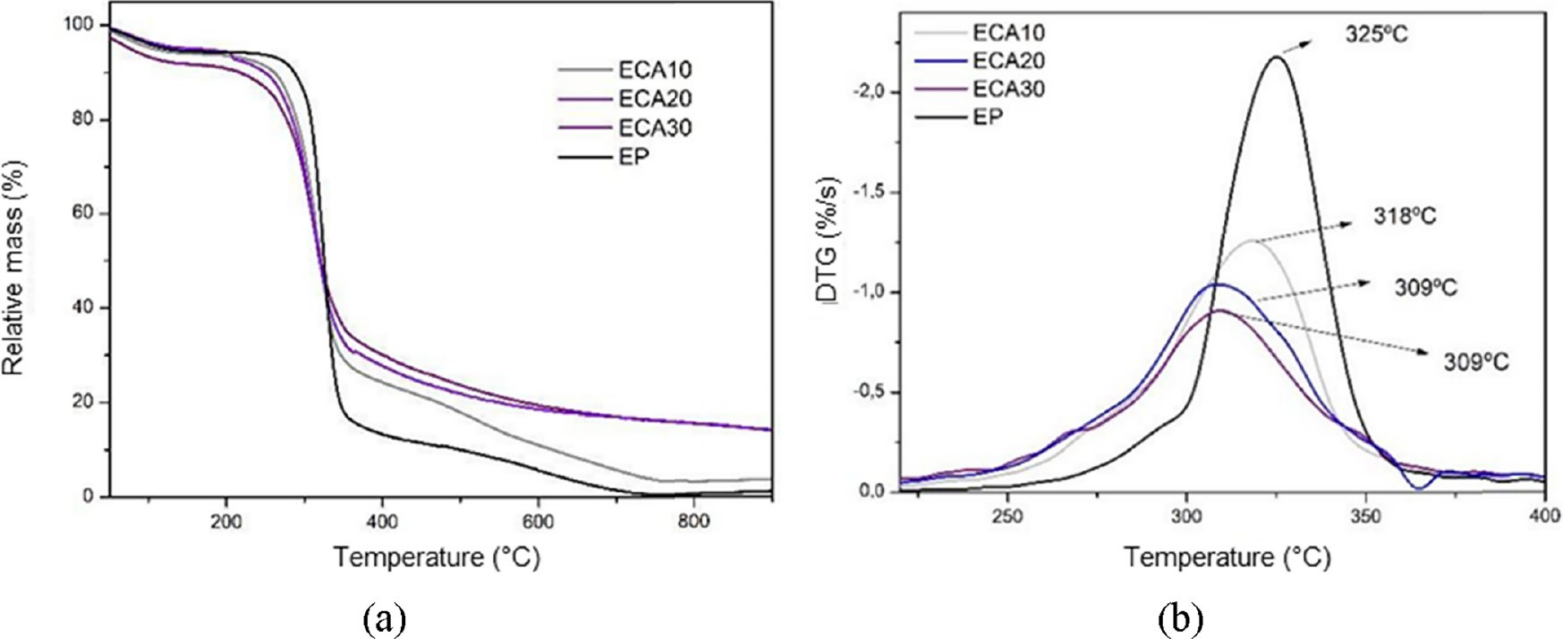
Thermogravimetric analysis (a) and first derivative of mass loss (b) of standard starch foams (EP) and incorporated with different levels of grape skin residue: ECA10 (10 wt %), ECA20 (20 wt %), and ECA30 (30 wt %).

**Figure 11:**
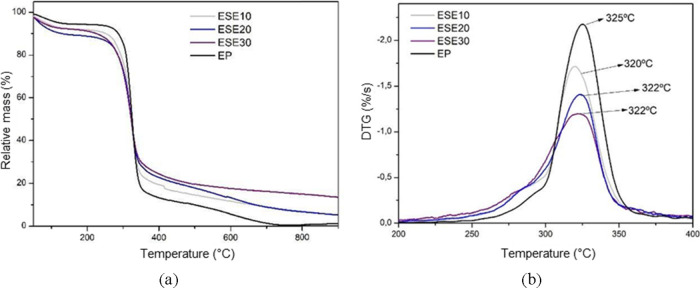
Thermogravimetric analysis (a) and first derivative of mass loss (b) of standard starch foams (EP) and incorporated with different levels of grape seed residue: ESE10 (10 wt %), ESE20 (20 wt %), and ESE30 (30 wt %).

**Figure 12:**
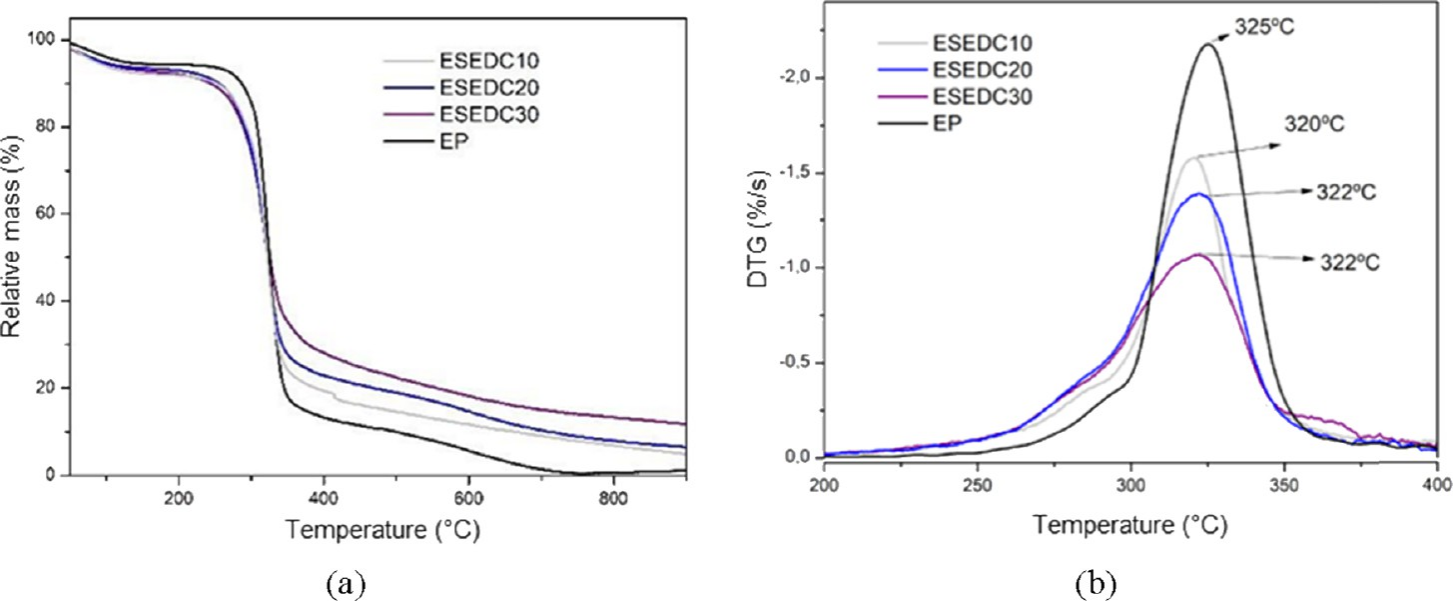
Thermogravimetric (a) and first derivative of mass loss (b) of standard starch foams (EP) and incorporated with different levels of degreased seed residue with grape skin fragments: ESEDC10 (10 wt %), ESEDC20 (20 wt %), and ESEDC30 (30 wt %).

Figures 9, 10, 11, and 12 illustrate the TG and DTG profiles of the samples obtained from cassava starch foam, both in the standard formulation and with the addition of CA, SE, and SEDC residues.

The starch used as the raw material for all formulations tested exhibited mass losses of 10.5 % in the temperature range of 150–310 °C. In the 310–400 °C range, the mass loss was higher at 60.6 %, with a residual mass of 5.7 % observed at 900 °C.

The control formulation foam (EP) showed a mass loss of 16.8 % in the 150–310 °C range and a mass loss of 64.5 % in the temperature range of 310–400 °C, with a residual mass of 1.2 % at 900 °C. Dony and Berzin, evaluating starch-based thermopolymers, reported similar thermogravimetry curves, with mass losses in the range of 80 % at 400 °C.^32^

The DTG profiles for the control sample (EP) and the starch showed the same maximum degradation rate at 325 °C. There was no noticeable difference in thermal stability between the EP formulation and the cassava starch used as the raw material. The TG and DTG curves for foams produced with different formulations containing CA, SE, and SEDC residues are shown in Figures 10, 11, and 12, respectively.

The samples obtained with the addition of CA residue showed mass losses in the range of 150–310 °C, with percentages of 31.1 %, 35.9 %, and 36.1% for samples ECA10, ECA20, and ECA30, respectively. In the 310–400 °C temperature range, the mass losses were 38.70 %, 31.43 %, and 28.87 % for samples ECA10, ECA20, and ECA30, respectively. The residual mass percentages at 900 °C were 3.73 %, 14.15%, and 14.20 % for samples ECA10, ECA20, and ECA30, respectively.

The DTG profiles for foams with added CA residue demonstrate a reduction in thermal stability, with a peak temperature of 309 °C observed for both the ECA20 and ECA30 samples. This reduction in thermal stability may be attributed to the presence of thermolabile and volatile compounds in the CA residue, which is intensified by higher concentrations.^11^

The samples containing SE residue exhibited mass losses in the temperature range of 150–310 °C, with 23.5 %, 24.5 %, and 28.3 % for samples ESE10, ESE20, and ESE30, respectively. In the 310–400 °C temperature range, the mass losses were 49.4 %, 42.8 %, and 39.6 % for samples ESE10, ESE20, and ESE30, respectively. The residual mass percentages at 900 °C were 4.92 %, 5.20 %, and 13.40 % for samples ESE10, ESE20, and ESE30, respectively.

The DTG profiles for the foams with the addition of the SE residue demonstrate minimal reduction in thermal stability. Notably, samples ESE30 and ESE20 exhibited the same peak temperature of 322 °C.

The samples containing SEDC residue showed mass losses ranging from 150 to 310 °C of 24.3 %, 27.7 %, and 28.0 % for samples ESEDC10, ESEDC20, and ESEDC30, respectively. In the 310–400 °C temperature range, the mass losses were 41.3 %, 39.6 %, and 35.8 % for samples ESEDC10, ESEDC20, and ESEDC30, respectively. The residual mass percentages at 900 °C were 4.0 %, 6.6 %, and 11.8 % for samples ESEDC10, ESEDC20, and ESEDC30, respectively.

The DTG profiles for the foams with the addition of SEDC residue indicated that there was practically no reduction in the thermal stability of the samples. For samples ESEDC30 and ESEDC20, the peak temperature observed was 322 °C.

A trend was noted in all foam samples obtained with the addition of waste: as the percentage of residue increased, so did the mass loss in the temperature range of 150 - 310 °C. This phenomenon may be related to the decomposition of hemicellulose present in the waste.^32,34^ Furthermore, the foam samples containing CA residue showed the highest percentage of mass loss in this range compared to those with SE and SEDC residues. This finding is consistent with the fact that the CA residue has the highest hemicellulose content among the residues evaluated.^11^

For samples with SE and SEDC residues, a greater mass loss was observed in the temperature range of 310–400 °C when CA residue was added. This phenomenon may be associated with the degradation of the less stable fraction of lignin and cellulose, particularly given that SE and SEDC residues have higher cellulose and lignin content compared to CA.^11,34,35^

The samples with SE and SEDC residues showed similar thermal stability, with stability values higher than those with addition of the CA residue. Such behavior aligns with the observation that the CA residue presented the lowest thermal stability compared to the formulations containing SEDC and SE residues.

### 3.7 FTIR

**Figure 13:**
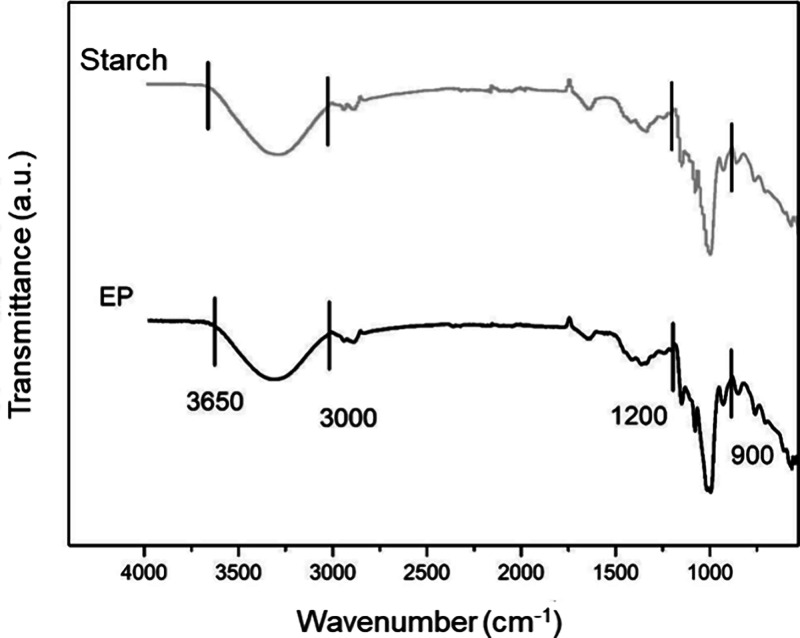
Cassava starch and standard starch foam (EP) FTIR spectra.

**Figure 14:**
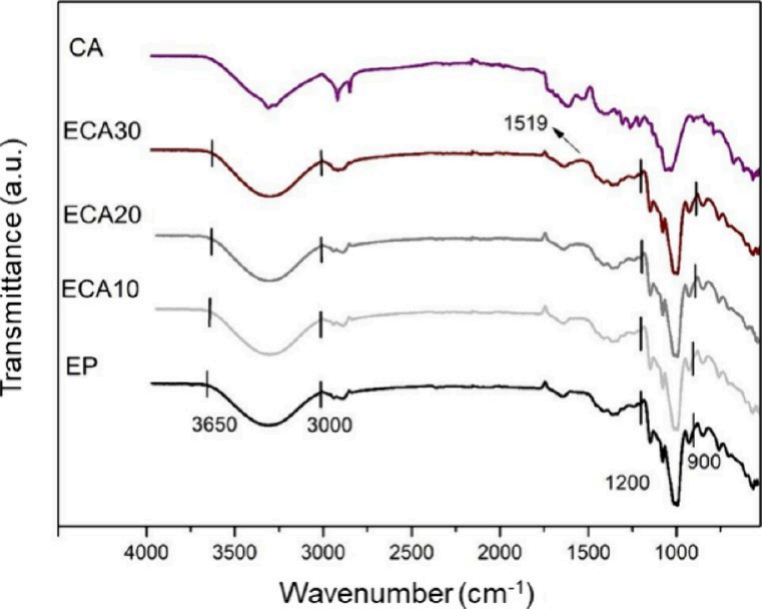
FTIR spectra of grape skin residue (CA), standard starch foams (EP), and incorporated with different levels of grape skin residue: ECA10 (10 wt %), ECA20 (20 wt %), and ECA30 (30 wt %).

**Figure 15:**
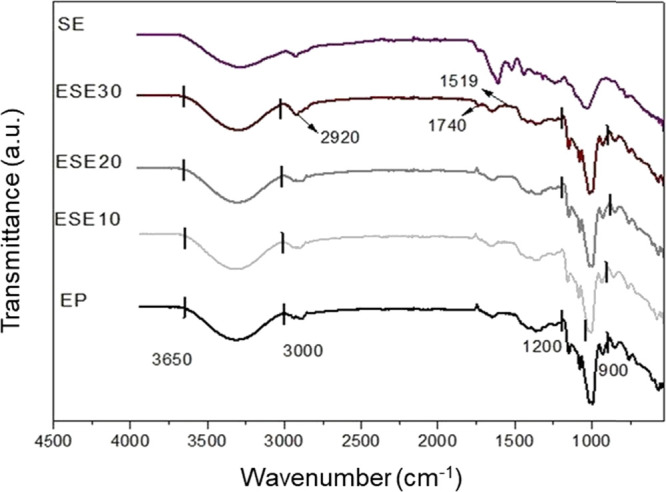
FTIR spectra of grape seed residue (SE), standard starch foams (EP), and incorporated with different levels of grape seed residue: ESE10 (10 wt %), ESE20 (20 wt %), and ESE30 (30 wt %).

**Figure 16:**
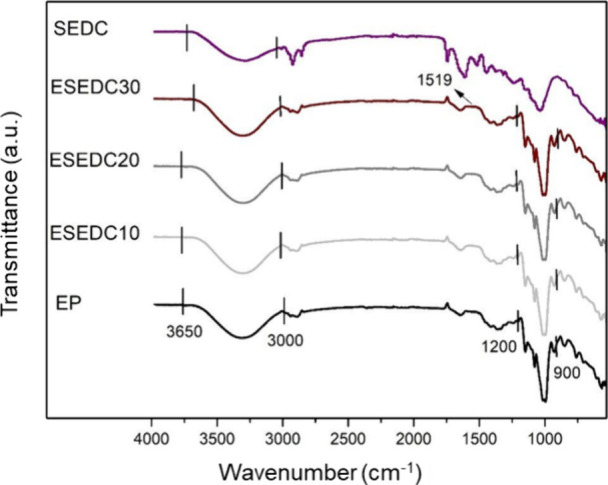
FTIR spectra of degreased seed residue with grape skin fragments (SEDC), of standard starch foams (EP), and incorporated with different contents of defatted seed residue with grape skin fragments: ESEDC10 (10 wt %), ESEDC20 (20 wt %), and ESEDC30 (30 wt %).

FTIR analysis was performed on all foam samples. Figures 13, 14, 15, and 16 show the infrared absorption spectra obtained for foam samples based on the control formulation (EP) and those with the addition of CA, SE, and SEDC residues. The FTIR analysis was carried out to evaluate possible interactions between cassava starch and the residues incorporated into the foam formulation.

Analyzing the spectra, it is observed that all samples exhibited vibrational bands between 3000 - 3650 cm^–1^. These bands can be related to stretching vibrations of the hydroxyl groups (−OH), featuring the presence of acetal (COC) and hydroxyl (−OH) groups associated with the starch molecules present in cassava starch.^10,36,37^ The OH stretching vibrations of the absorbed water in the starch refer to stretching the vibrational complex associated with free and bound intra- and intermolecular hydroxyl groups.^3,24^

Furthermore, more intense bands are observed in the 900–1200 cm^–1^ range, attributed to the COC bonds’ vibrations. This is characteristic of starch and other polysaccharides, with greater intensity indicating interactions between the formulation components.^38^

In the ECA30, ESE30, and ESEDC30 samples, a slight difference in the band intensity at 1519 cm^–1^ can be noticed compared to the EP sample. This difference can be attributed to the stretching of the CC bond in aromatic compounds, which are related to phenolic compounds.^36^

The spectrum of the ESE30 sample reveals minor differences in the bands at 1740 cm^–1^ and 2920 cm^–1^ when compared to the EP sample. These differences are related to the presence of lignins and hydrocarbon chains of lipids, compounds absent in the tray composed only of starch.^39,40^

Overall, the bands observed in the EP sample are similar to those in the foam samples containing CA, SE, and SEDC residues.^11^ The leading vibrational bands, between 3000 - 3650 cm^–1^ and between 900 - 1200 cm^–1^, were also reported by Bergel et al.^41^ and Engel et al.^10^ in their studies of starch-based foams, respectively. It is also important to note that, based on the characteristics of the spectra obtained, no significant chemical reactions or important heat-induced changes occurred in the foams.

### 3.8 Tensile Test

The tensile test results of foam samples incorporating CA, SE, and SEDC residues are presented in Table 6.

**Table 6:** Mechanical Properties of Foams Based on Cassava Starch and Incorporated with Different Residue Levels: EP (Standard Starch Foam), with the Addition of Grape Skin Residue: ECA10 (10 wt.%), ECA20 (20 wt.%), and ECA30 (30 wt.%); with the Addition of Grape Seed Residue: ESE10 (10 wt.%), ESE20 (20 wt.%), and ESE30 (30 wt.%); and with the Addition of Defatted Seed Residue with Grape Skin Fragments: ESEDC10 (10 wt.%), ESEDC20 (20 wt %), and ESEDC30 (30 wt.%)^a^

sample	rupture tension (MPa)	elongation at break(%)	modulus of elasticity (MPa)
EP	1.01 ± 0.08a	2.8 ± 0.2a	38.3 ± 2.2a
ECA10	0.42 ± 0.02d	2.2 ± 0.3a	15.7 ± 1.3c
ECA20	0.34 ± 0.01e	1.5 ± 0.1c	13.8 ± 1.0c
ECA30	0.17 ± 0.02g	1.2 ± 0.1d	9.2 ± 0.9d
ESE10	0.63 ± 0.05b	2.5 ± 0.3a	23.4 ± 1.7b
ESE20	0.48 ± 0.01c	2.0 ± 0.1a	16.5 ± 1.1c
ESE30	0.36 ± 0.03e	1.4 ± 0.1c	14.6 ± 1.6c
ESEDC10	0.59 ± 0.02b	2.4 ± 0.2a	20.2 ± 2.1b
ESEDC20	0.42 ± 0.01d	2.2 ± 0.3a	15.5 ± 1.3c
ESEDC30	0.24 ± 0.04f	1.7 ± 0.1b	13.1 ± 1.2c

a Means in the same column with different lowercase letters show significant differences using the Tukey test at a 5 % probability of error (*p* < 0.05).

The tensile strength of the samples ranged from 0.17 MPa (ECA30) to 1.01 MPa (EP), with a significant difference between these values (*p* < 0.05). The results of the tensile tests indicate a decrease in rupture tension, elongation, and modulus of elasticity as the concentration of residues in the formulation increased. The reduction in these properties can be related to the lower apparent density, which leads to larger cells in the internal structure compared to the control sample (EP) and weakening the structure as the residue content increases.

Furthermore, increasing the amount of fiber can interfere with expandability, causing discontinuities in the starch matrix. Consequently, the added residues may introduce defects in the polymeric structure, leading to points of tension accumulation. This accumulation facilitates material rupture at lower tensions, resulting in foams with lower resistance to rupture.^3,9^

Foam samples containing CA residue exhibited the lowest rupture stress values, varying in the 0.17–0.42 MPa range. A significant statistical difference was noted with variations in the concentration of the added residue. These values correlate with the lower apparent density observed in foams with CA residue. Since this residue has the highest water absorption capacity, it resulted in suspensions with higher water contents than foams with SE and SEDC residues.

The water in the formulation is eliminated as vapor during the thermal expansion process, promoting a larger number of internal voids in the foam structure. This phenomenon induces greater structural fragility, especially when the proportion of starch in the formulation is smaller.^10^

The foam samples containing SE residue exhibited stress values varying in the 0.36–0.63 MPa range. A statistically significant difference (*p* < 0.05) was observed between these values as the percentage of residue added to the formulation increased.

In contrast, the foam samples with SEDC residue showed rupture stress values ranging from 0.24 - 0.59 MPa, with a significant difference (*p* < 0.05) noted as the percentage of residue increased.

The highest rupture stress values recorded were for the formulations ESE10 (0.63 MPa) and ESEDC10 (0.59 MPa), with no significant difference (*p* < 0.05) between them. These values may be related to the higher apparent densities of these foams compared to the other samples.

The elastic moduli of the samples ranged from 9.2 MPa (ECA30) to 38.3 MPa (EP), with a significant difference (*p* < 0.05) between these values. The incorporation of greater amounts of waste resulted in a decrease in the elastic modulus, making the samples more fragile. Samples ECA10, ECA20, ESE20, ESE30, ESEDC20, and ESEDC30 did not show a significant difference (*p* < 0.05) in terms of elastic modulus. The best results, although lower than those of the control sample (EP), were obtained for the ESE10 and ESEDC10 samples, which also showed no significant difference (*p* < 0.05) between them.

The tensile test results for foam samples with waste were lower than the reference values for commercial PS foams, which had a rupture stress of 0.75 MPa, elongation of 4.9 %, and modulus of elasticity of 18.1 MPa.^3,9^

Cabanillas et al.^9^ reported that the tensile strength of starch foams increased to 0.83 MPa by adding 5 wt.% of pineapple peel fibers, a value comparable to that of PS foams. The authors attributed this increase in tensile strength to the reinforcement effect resulting from the interfacial interaction between the fiber and the starch matrix. This phenomenon may involve stress transfer from the starch matrix to the fibers during foam deformation, contributing to enhanced mechanical resistance. However, the authors also noted that higher fiber ratios (starch:fiber ratios of 85:15 and 90:10) significantly reduced the tensile strength due to agglomeration and discontinuity of fibers in the polymer matrix. Similar findings were obtained with cassava starch reinforced with 4.0 wt.% chitosan fiber, which had a tensile strength of 0.74 MPa.^42^

Engel et al. reported the mechanical properties of foams based on cassava starch combined with grape stems developed from an optimized formulation (81.6 wt.% cassava starch, 13.6 wt.% glycerol, and 18.4 wt.% grape stems), noting a rupture stress of 0.48 MPa, an elongation of 1.6 %, and an elastic modulus of 41.0 MPa.^43^ Strategies to address the weaker mechanical properties may include increasing the plasticizer content or reducing the particle size of the residues to allow for better dispersion, facilitating the interaction between the starch matrix and the particles, and reducing punctual tension accumulation.^10−43^

### 3.9 Antimicrobial Activity by Survival Determination

Initially, the plating test was carried out using the highest concentrations of the residues. The samples ECA30, ESE30, ESEDC30, and the control sample (EP) were evaluated to determine the growth sensitivity of the yeast *Candida albicans* CA01 and the bacteria *Escherichia coli* INCQS00033 and *Staphylococcus aureus* INCQS00015. The results are presented in Table 7.

**Table 7:** Microbial Activity by Determining the Survival of Foams Based on Cassava Starch and Incorporated with Different Residue Levels: EP (Standard Starch Foam), with the Addition of Grape Skin Residue: ECA30 (30 wt.%); with the Addition of Grape Seed Residue: ESE30 (30 wt.%); and with the Addition of Defatted Seed Residue with Grape Skin Fragments: ESEDC30 (30 wt.%)^a^

microbial activity – growth percentage			
sample	Candida albicans CA01	Escherichia coli INCQS00033	Staphylococcus aureus INCQS00015
EP	100.00 ± 20.60a,b	100.00 ± 13.63b	100.00 ± 22.22a
ECA30	112.66 ± 10.13a	0.93 ± 0.29c	34.92 ± 9.70b
ESE30	79.97 ± 6.36b	113.27 ± 9.59a,b	117.46 ± 18.33a
ESEDC30	89.13 ± 9.07b	128.91 ± 14.12a	109.52 ± 19.40a

a Means in the same column with different lowercase letters show significant differences using the Tukey test at a 5 % probability of error (*p* < 0.05).

Adding CA residues to the foams did not reduce the percentage of growth of the *C. albicans* yeast compared to the control (EP), as no significant difference was observed in the results. On the other hand, a modest reduction in the growth of *C. albicans* was noted with the addition of SE and SEDC residues.

Regarding the ESE30 and ESEDC30 samples, there was no reduction in the growth of *E. coli* and *S. aureus* bacteria compared to standard foam (EP).

The ECA30 sample showed a 99 % reduction in the growth of *E. coli* and a 65 % reduction in the growth percentage of *S. aureus* concerning the EP sample. Due to these results, the test was repeated with samples ECA10 and ECA20, and the results are presented in Table 8.

**Table 8:** Microbial Activity by Determining the Survival of Microorganisms Exposed to Foams Based on Cassava Starch and Incorporated with Different Residue Levels: EP (Standard Starch Foam), with the Addition of Grape Skin Residue: ECA10 (10 wt.%), ECA20 (20 wt.%), and ECA30 (30 wt.%)^a^

microbial activity – growth percentage		
sample	Escherichia coli INCQS00033	*Staphylococcus aureus* INCQS00015
ECA10	42.00 ± 25.71a	201.21 ± 36.32b
ECA20	3.33 ± 2.73b	297.98 ± 34.26a
ECA30	0.93 ± 0.29b	34.92 ± 9.7c

a Means in the same column with different lowercase letters show significant differences using the Tukey test at a 5 % probability of error (*p* < 0.05).

The results indicated that 30 wt % CA residue concentration provided the highest inhibition of *S. aureus* growth compared to the other samples. In contrast, at the concentrations of 10 wt % and 20 wt %, there was an observed increase in bacterial growth.

Samples ECA20 and ECA30 showed higher inhibition percentages against *E. coli*, with no significant difference between these values. Notably, both ECA30 and ECA20 contained the highest levels of anthocyanins, which may play a role in inhibiting the growth of these bacteria.

Anthocyanins and other phenolic compounds are widely recognized for their antimicrobial activity.^44^ Studies by Salamon et al.^45^ and Dong et al.^46^ reported a potent antimicrobial effect of different types of anthocyanins against bacteria such as the bacteria *S. aureus*, *E. coli*, *E. faecalis*, and *S. pyogenes*, as well as the yeast *C. albicans*. Additionally, these compounds demonstrated inhibitory and cytotoxic activity against cancer cells.

According to García-Lomillo and González-San José, grape pomace residue possesses unique characteristics, being capable of inhibiting different microbiological agents and chemical reactions.^47^ This ability allows for a reduction in the use of synthetic materials, preservatives, and food antioxidants without compromising the stability and quality of the final product.

### 3.10 Biodegradation Test

**Figure 17:**
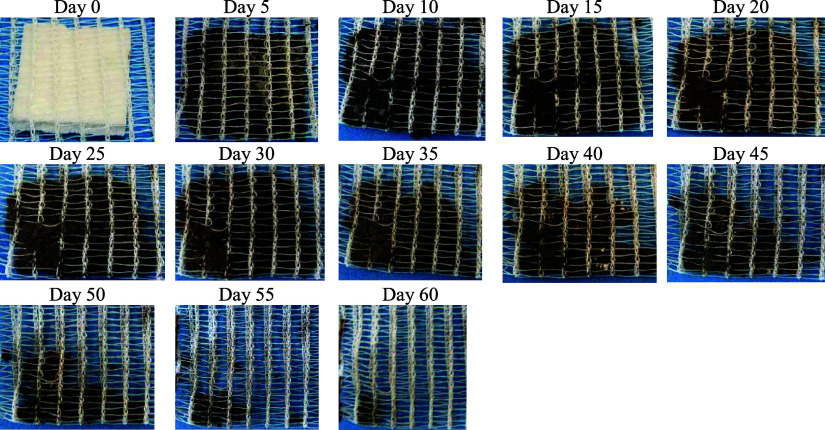
Images of the degradation of cassava starch foam from the standard sample (EP) over 60 days.

**Figure 18:**
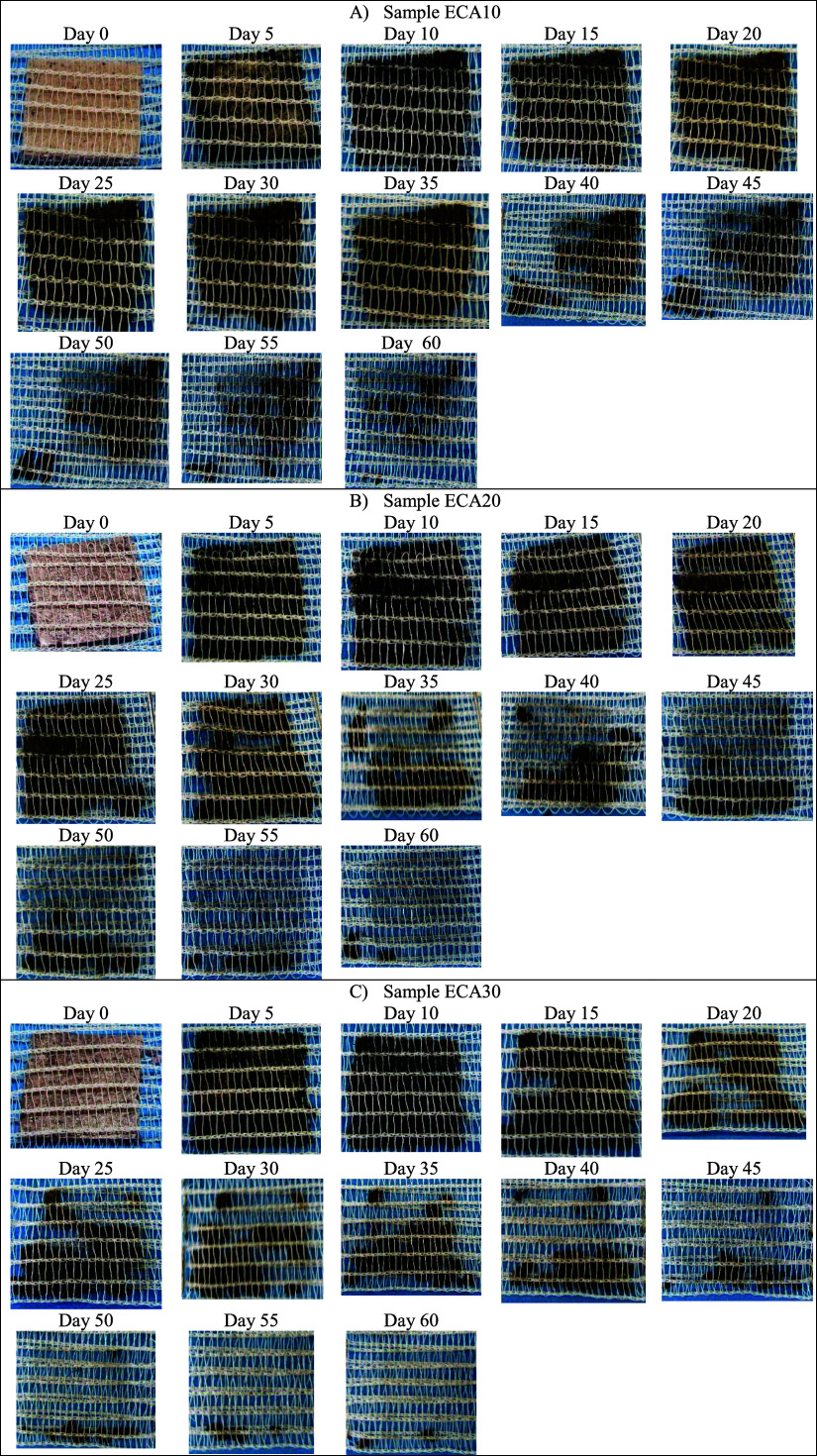
Images of the degradation of cassava starch foam with the incorporation of grape skin residue (CA): (a) ECA10 (10 wt %); (b) ECA20 (20 wt %); (c) ACE30 (30 wt %) over 60 days.

**Figure 19:**
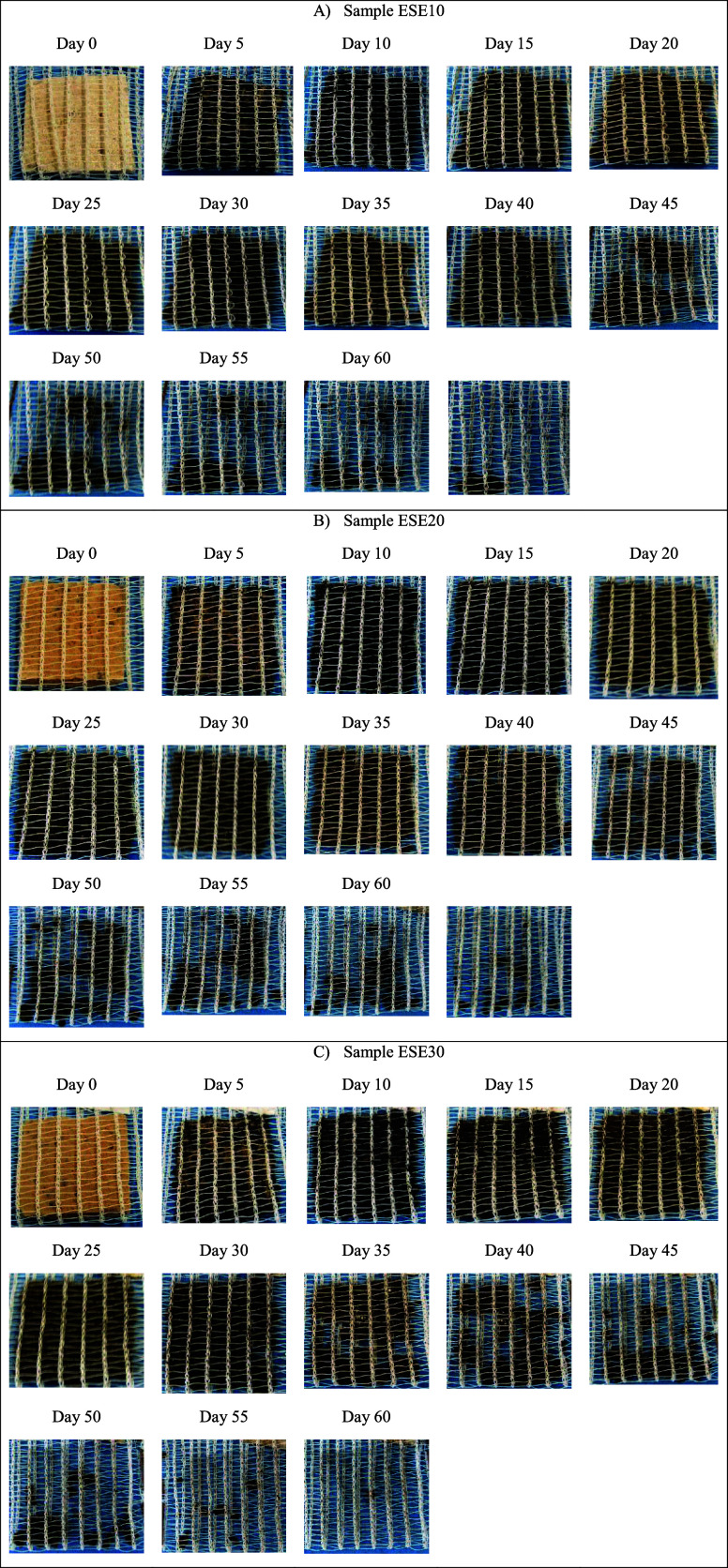
Images of the degradation of cassava starch foam with the incorporation of grape seed residue (SE): (a) ESE10 (10 wt %); (b) ESE20 (20 wt %); (c) ESE30 (30 wt %) over 60 days.

**Figure 20:**
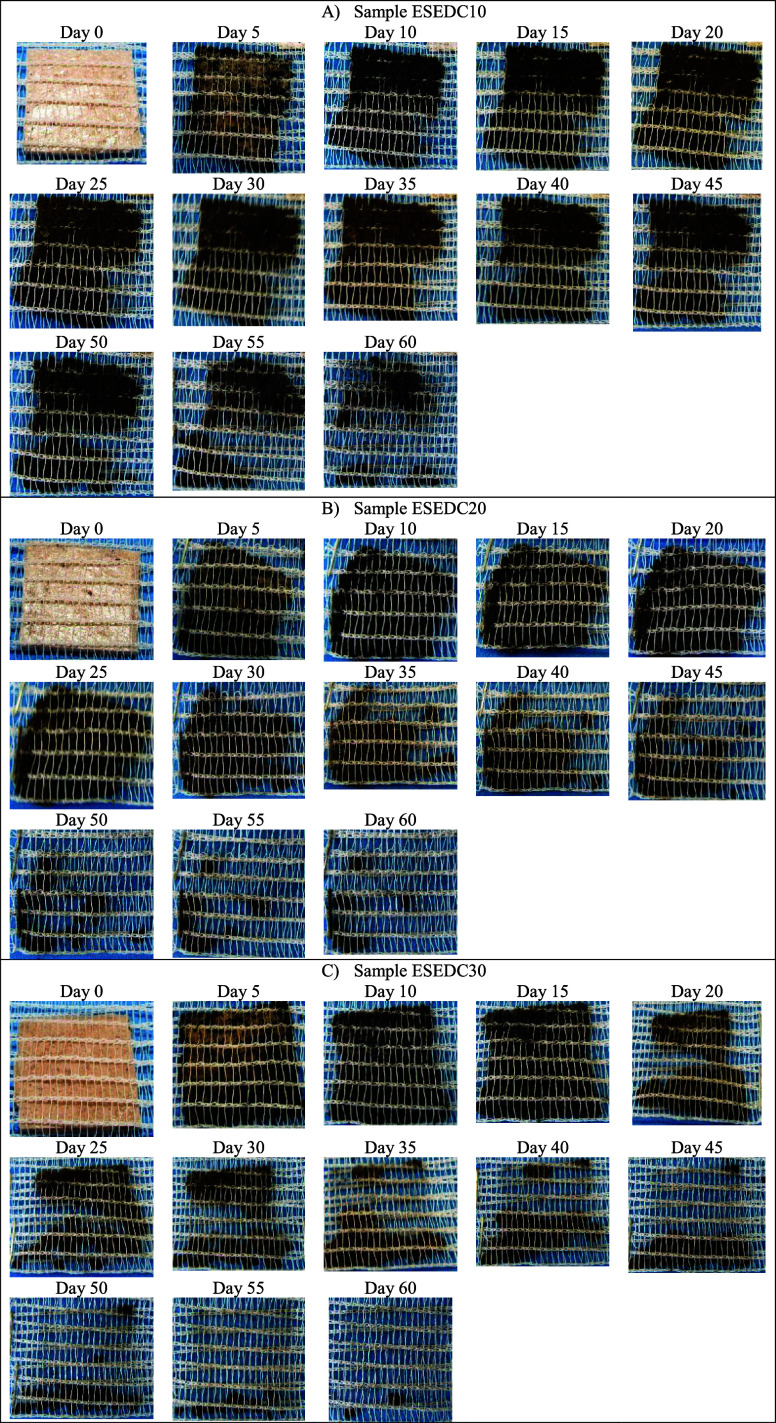
Images of the degradation of cassava starch foam with the incorporation of defatted grape seed residue with grape skin fragments (SEDC): (a) ESEDC10 (10 wt %); (b) ESEDC20 (20 wt %); and (c) ESEDC30 (30 wt %) over 60 days.

The results obtained from the biodegradation test of cassava starch foams from the standard sample (EP) and with the addition of CA, SEDC, and SE residues can be seen in Figures 17 to 20, with monitoring carried out for 60 days.

According to Figure 17, it can be seen that a gradual decomposition of the material occurred, with the structure beginning to disintegrate after 30 days. The sample degraded nearly entirely at the end of the 60-day test period. Nevoralová et al. reported that thermoplastic polymers based on starch and glycerol (70:30) degraded in approximately 45 days when exposed to soil.^48^

Visual records relating to foams containing CA residue are compiled in Figure 18.

It can be seen from Figure 18 that the increase in the CA residue content added to the formulation enhanced the biodegradation process, as the CA10 sample remained intact until 35 days after the start of the test, while the CA20 sample began to deteriorate and lose integrity in 25 days. The CA30 sample started disintegrating in the first 15 to 20 days. Thus, it can be considered that grape skin (CA waste) can be a possible substrate for decomposing microorganisms in the soil, accelerating the biodegradation process.

As noted by Sirohi et al., grape processing byproducts are a source of bioactive compounds, nutrients and minerals for the decomposing microorganisms in the soil.^49^ Furthermore, as commented by Zanini et al.,^11^ grape skin is composed of an important portion of hemicellulose, which is easily decomposed and processed by the soil microbiota, which may explain the faster biodegradation of foams containing higher levels of CA residue.

The images referring to the foams containing the SE residue are compiled in Figure 19.

Concerning foams containing grape seed residue (SE), it is noted that, regardless of the levels of residue added, all formulations began to disintegrate between 30 and 40 days after the beginning of exposure to the soil, indicating a behavior similar to standard starch foam (EP) and resistance to degradation slightly greater than that of foams containing CA residue.

The presence of seed oil, even at low levels, may have influenced the ability of microorganisms to adhere and decompose the material or may have affected the moisture absorption capacity of the material since materials with lower seed oil activity water are more difficult to biodegrade.^50^ Possible antioxidant effects and inhibitory effects of grape seed oil on soil-decomposing microorganisms may also occur, delaying the biodegradation process of foams containing this residue.^51^

The images referring to the foams containing the SE residue are compiled in Figure 20.

It can be observed that the foams containing higher levels of SEDC residue showed faster degradation since, for sample SEDC10, the disintegration of the structure started in around 45 days, for sample SEDC20, it started in around 30 days and, for sample SEDC30, it started in 20 days.

The lower oil content, with the presence of seed and grape skin fragments composed mainly of hemicellulose and cellulose,^11,49^ may have facilitated the action of decomposing microorganisms, which would explain the increase in the biodegradation rate of the foams with the increase in the amount of residue added to the formulation. Thus, the SEDC residue, due to its composition, probably facilitated the biodegradation process of the foams compared to the control (EP).

In any case, residues from grape processing, regardless of plant origin (seed, skin, stem, rachis), have a high potential for biodegradation and are a source of minerals, bioactive compounds, and precursor molecules for the metabolism of microorganisms and superior beings, which explains the enhancement of the biodegradation capacity of foams with the addition of CA, SE and SEDC residues to the formulations.^52,53^

## 4 Conclusions

Incorporating CA, SE, and SEDC residues into starch foams showed potential for developing active packaging with properties of antioxidant capacity. Foams with SEDC residue exhibited the highest antioxidant capacity and higher levels of flavonoids and total phenolics. Regarding thermal stability, adding CA, SE, and SEDC residues decreased the thermal stability of the foams compared to the control sample. In the spectra obtained, it was possible to identify characteristic bands corresponding to the added residues. Including these residues enhanced the biodegradation capacity of the foams, which disintegrated within 20 to 40 days and completely degraded in approximately 60 days. However, the mechanical properties of the foams with CA, SE, and SEDC residues were lower than those of both the control sample and EPS. In general, foams with SE and SEDC residues showed better results when compared to foams with CA residue. Regarding antimicrobial activity, foams containing CA residue promoted partial inhibition of *S. aureus* and *E. coli* development. Further research is needed to optimize the formulations, particularly concerning water absorption capacity and mechanical resistance for commercial applications.

## References

[ref1] Jorge N. Embalagens para alimentos. Cultura Acadêmica; UNESP, 2013.

[ref2] Soykeabkaew N.; Thanomsilp C.; Suwantong O. A review: Starch-based composite foams. Comp. A Appl. Sci. Manufact. 2015, 78, 246–263. 10.1016/j.compositesa.2015.08.014.

[ref3] Machado C. M.; Benelli P.; Tessaro I. C. Sesame cake incorporation on cassava starch foams for packaging use. Ind. Crops Prod. 2017, 102, 115–121. 10.1016/j.indcrop.2017.03.007.

[ref4] Salwa H. N.; et al. Green biocomposites for food packaging. Int. J. Rec. Technol. Eng. 2019, 8, 450–459. 10.35940/ijrte.B1088.0782S419.

[ref5] Mendes A. C.; Pedersen G. A. Perspectives on sustainable food packaging: is bio-based plastics a solution?. Trends Food Sci. Technol. 2021, 112, 839–846. 10.1016/j.tifs.2021.03.049.

[ref6] Otto S.; Strenger M.; Maier-Nöth A.; Schmid M.; et al. Food packaging and sustainability – Consumer perception vs. correlated scientific facts: A review. J. Cleaner Prod. 2021, 298, 126733 10.1016/j.jclepro.2021.126733.

[ref7] Cheng H.; et al. Starch-based biodegradable packaging materials: A review of their preparation, characterization and diverse applications in the food industry. Trends Food Sci. Technol. 2021, 114, 70–82. 10.1016/j.tifs.2021.05.017.

[ref8] Lopez-Gil A.; et al. Strategies to Improve the Mechanical Properties of Starch-Based Materials: Plasticization and Natural Fibers Reinforcement. Polím. Ciênc. Tecnol. 2014, 24, 36–42. 10.4322/polimeros.2014.054.

[ref9] Cabanillas A.; Nuñez J.; Cruz-Tirado J. P.; Vejarano R.; Tapia-Blácido D. R.; Arteaga H.; Siche R.; et al. Pineapple shell fiber as reinforcement in cassava starch foam trays. Polym. Polym. Compos. 2019, 27, 496–506. 10.1177/0967391119848187.

[ref10] Engel J. B.; Ambrosi A.; Tessaro I. C. Development of biodegradable starch-based foams incorporated with grape stalks for food packaging. Carbohydr. Polym. 2019, 225, 115234 10.1016/j.carbpol.2019.115234. 31521283

[ref11] Zanini M.; Silvestre W. P.; Baldasso C.; Tessaro I. C. Valorization of wastes generated in organic grape processing. Braz. Arch. Biol. Technol. 2024, 67, e24230183 10.1590/1678-4324-2024230183.

[ref12] Baratter M.; Weschenfelder E. F.; Stoffel F.; Zeni M.; Piemolini-Barreto L. T. Analysis and Evaluation of Cassava Starch-Based Biodegradable Trays as an Alternative Packaging to Fresh Strawberry (*Fragaria ananassa* Cv San Andreas). Food Nutr. J. 2017, 3, 126 10.29011/2575-7091.100026.

[ref13] Shogren R. L.; et al. Structure and morphology of baked starch foams. Polymer. 1998, 39, 6649–6655. 10.1016/S0032-3861(97)10303-2.

[ref14] Instituto Adolfo Lutz (IAL) Métodos físico-químicos para análise de alimentos. Instituto Adolfo Lutz: São Paulo, 2018.

[ref15] Associação Brasileira De Normas Técnicas (ABNT) Papel e cartão: Determinação da capacidade de absorção de água - Método de Cobb; NBR NM ISO 535:1999, 1999.

[ref16] Singleton V. L.; Rossi J. A. Colorimetry of Total Phenolics with Phosphomolybdic-Phosphotungstic Acid Reagents. Am. J. Enol. Viticult. 1965, 16, 144–158. 10.5344/ajev.1965.16.3.144.

[ref17] Lima M. D. S.; et al. Phenolic compounds, organic acids and antioxidant activity of grape juices produced in industrial scale by different processes of maceration. Food Chem. 2015, 188, 384–392. 10.1016/j.foodchem.2015.04.014. 26041208

[ref18] Matic P.; Sabljic M.; Jakobek L. Validation of Spectrophotometric Methods for the Determination of Total Polyphenol and Total Flavonoid Content. J. AOAC Int. 2017, 100, 1795–1803. 10.5740/jaoacint.17-0066. 28730980

[ref19] Association of Official Analytical Chemists (AOAC) Official Methods of Analysis of AOAC International. 18 ed.; AOAC: MD, 2005.

[ref20] Yamaguchi T.; et al. HPLC method for evaluation of the free radical-scavenging activity of foods by using 1,1-diphenyl-2-picrylhydrazyl. Biosci. Biotechnol. Biochem. 1998, 62, 1201–1204. 10.1271/bbb.62.1201. 9692204

[ref21] Rufino M. S. M. Metodologia científica: determinação da atividade antioxidante total em frutas pela captura do radical livre DPPH. Embrapa Agroindústria Tropical: Fortaleza, 2007.

[ref22] American Society for Testing and Materials (ASTM) Standard test method for tensile properties of plastics (D 638–02); ASTM international 2003.

[ref23] Medina Jaramillo C.; Gutiérrez T. J.; Goyanes S.; Bernal C.; Famá L. Biodegradability and plasticizing effect of yerba mate extract on cassava starch edible films. Carbohydr. Polym. 2016, 151, 150–159. 10.1016/j.carbpol.2016.05.025. 27474554

[ref24] Vercelheze A. E. S.; et al. Properties of baked foams based on cassava starch, sugarcane bagasse fibers and montmorillonite. Carbohydr. Polym. 2012, 87, 1302–1310. 10.1016/j.carbpol.2011.09.016.

[ref25] Jarpa-Parra M.; Chen L. Applications of plant polymer-based solid foams: Current trends in the food industry. Appl. Sci. 2021, 11, 9605 10.3390/app11209605.

[ref26] Cruz-Tirado J. P.; Barros Ferreira R. S.; Lizárraga E.; Tapia-Blácido D. R.; Silva N. C. C.; Angelats-Silva L.; Siche R.; et al. Bioactive Andean sweet potato starch-based foam incorporated with oregano or thyme essential oil. Food Packag. Shelf Life. 2020, 23, 100457–13. 10.1016/j.fpsl.2019.100457.

[ref27] Kaisangsri N.; Kerdchoechuen O.; Laohakunjit N. Characterization of cassava starch based foam blended with plant proteins, kraft fiber, and palm oil. Carbohydr. Polym. 2014, 110, 70–77. 10.1016/j.carbpol.2014.03.067. 24906730

[ref28] Berger C.; et al. Production of sustainable polymeric composites using grape pomace biomass. Biomass Conv. Bioref. 2022, 12, 5869–5880. 10.1007/s13399-020-00966-w.

[ref29] Cejudo-Bastante C.; Arjona-Mudarra P.; Fernández-Ponce M. T.; Casas L.; Mantell C.; Martínez de la Ossa E. J.; Pereyra C.; et al. Application of a natural antioxidant from grape pomace extract in the development of bioactive jute fibers for food packaging. Antioxidants. 2021, 10, 216–15. 10.3390/antiox10020216. 33540565 PMC7912872

[ref30] Gasiński A.; et al. Application of white grape pomace in the brewing technology and its impact on the concentration of esters and alcohols, physicochemical parameters and antioxidative properties of the beer. Food Chem. 2022, 367, 130646 10.1016/j.foodchem.2021.130646. 34364146

[ref31] Nakov G.; Brandolini A.; Hidalgo A.; Ivanova N.; Stamatovska V.; Dimov I.; et al. Effect of grape pomace powder addition on chemical, nutritional and technological properties of cakes. LWT. 2020, 134, 109950–115. 10.1016/j.lwt.2020.109950.

[ref32] Dony P.; Berzin F. Thermogravimetric, Morphological and Infrared Analysis of Blends Involving Thermoplastic Starch and Poly(ethylene-co-methacrylic acid) and Its Ionomer Form. Molecules. 2023, 28, 4519 10.3390/molecules28114519. 37298994 PMC10254938

[ref33] Echegaray M.; Saffe M.; Palacios C.; Mazza G.; Rodriguez R. Thermogravimetric and Kinetic Analysis of Different Agro-Industrial Wastes Under Nitrogen Atmosphere. Int. J. Eng. Innov. Res. 2015, 4, 213–219. 10.1016/j.foodchem.2021.130646.

[ref34] Valente M.; Brillard A.; Schönnenbeck C.; Brilhac J. F. Investigation of grape marc combustion using thermogravimetric analysis. Kinetic modeling using an extended independent parallel reaction (EPR). Fuel Proces. Technol. 2015, 131, 297–303. 10.1016/j.fuproc.2014.10.034.

[ref35] D’Eusanio V.; Malferrari D.; Marchetti A.; Roncaglia F.; Tassi L. Waste By-Product of Grape Seed Oil Production: Chemical Characterization for Use as a Food and Feed Supplement. Life. 2023, 13, 326 10.3390/life13020326. 36836684 PMC9958947

[ref36] Dong X.; et al. Fractionation and structural characterization of polysaccharides derived from red grape pomace. Process Biochem. 2021, 109, 37–45. 10.1016/j.procbio.2021.06.022.

[ref37] Avérous L. Biodegradable Multiphase Systems Based on Plasticized Starch: A Review. J. Macromolec. Sci. 2004, C44, 231–274. 10.1081/MC-200029326.

[ref38] Wokadala O. C.; Emmambux N. M.; Ray S. S. Inducing PLA/starch compatibility through butyl-etherification of waxy and high amylose starch. Carbohydr. Polym. 2014, 112, 216–224. 10.1016/j.carbpol.2014.05.095. 25129738

[ref39] Lupoi J. S.; et al. Recent innovations in analytical methods for the qualitative and quantitative assessment of lignin. Ren. Sust. Energy Rev. 2015, 49, 871–906. 10.1016/j.rser.2015.04.091.

[ref40] Lucarini M.; et al. Grape seeds: Chromatographic profile of fatty acids and phenolic compounds and qualitative analysis by FTIR-ATR spectroscopy. Foods. 2020, 9, 10 10.3390/foods9010010. PMC7023066 31877706

[ref41] Bergel B. F.; et al. Effects of hydrophobized starches on thermoplastic starch foams made from potato starch. Carbohydr. Polym. 2018, 200, 106–114. 10.1016/j.carbpol.2018.07.047. 30177146

[ref42] Cruz-Tirado J. P.; et al. Biodegradable foam tray based on starches isolated from different Peruvian species. Int. J. Biol. Macromolec. 2019, 125, 800–807. 10.1016/j.ijbiomac.2018.12.111. 30557647

[ref43] Engel J. B.; Ambrosi A.; Tessaro I. C. Development of a Cassava Starch-Based Foam Incorporated with Grape Stalks Using an Experimental Design. J. Polym. Environ. 2019, 27, 2853–2866. 10.1007/s10924-019-01566-0. 31521283

[ref44] Ma Y.; Ding S.; Fei Y.; Liu G.; Jang H.; Fang J. Antimicrobial activity of anthocyanins and catechins against foodborne pathogens *Escherichia coli* and *Salmonella*. Food Contr. 2019, 106, 106712 10.1016/j.foodcont.2019.106712.

[ref45] Salamon I.; Şimşek Sezer E. N.; Kryvtsova M.; Labun P. Antiproliferative and Antimicrobial Activity of Anthocyanins from Berry Fruits after Their Isolation and Freeze-Drying. Appl. Sci. 2021, 11, 2096 10.3390/app11052096.

[ref46] Dong Y.; Yang C.; Zhong W.; Shu Y.; Zhang Y.; Yang D. Antibacterial effect and mechanism of anthocyanin from *Lycium ruthenicum* Murr. Front. Microbiol. 2022, 13, 974602 10.3389/fmicb.2022.974602. 36060738 PMC9437951

[ref47] García-Lomillo J.; González-Sanjosé M. L. Applications of Wine Pomace in the Food Industry: Approaches and Functions. Compr. Rev. Food Sci. Food Saf. 2017, 16, 3–22. 10.1111/1541-4337.12238. 33371551

[ref48] Nevoralová M.; Koutný M.; Ujčić A.; Horák P.; Kredatusová J.; Šerá J.; Růžek L.; Růžková M.; Krejčíková S.; Šlouf M.; Kruliš Z. Controlled biodegradability of functionalized thermoplastic starch based materials. Polym. Degrad. Stab. 2019, 170, 108995 10.1016/j.polymdegradstab.2019.108995.

[ref49] Sirohi R.; Tarafdar A.; Singh S.; Negi T.; Gaur V. K.; Gnansounou E.; Bharathiraja B. Green processing and biotechnological potential of grape pomace: Current trends and opportunities for sustainable biorefinery. Bioresour. Technol. 2020, 314, 123771 10.1016/j.biortech.2020.123771. 32653247

[ref50] Tanaka M.; Sato K.; Kitakami E.; Kobayashi S.; Hoshiba T.; Fukushima K. Design of biocompatible and biodegradable polymers based on intermediate water concept. Polym. J. 2015, 47, 114–121. 10.1038/pj.2014.129.

[ref51] Garavaglia J.; Markoski M. M.; Oliveira A.; Marcadenti A. Grape Seed Oil Compounds: Biological and Chemical Actions for Health. Nutr. Metab. Insights. 2016, 9, 10 10.3390/foods9010010. PMC4988453 27559299

[ref52] Baroi A. M.; Popitiu M.; Fierascu I.; Sărdărescu I. D.; Fierascu R. C. Grapevine Wastes: A Rich Source of Antioxidants and Other Biologically Active Compounds. Antioxidants. 2022, 11, 393 10.3390/antiox11020393. 35204275 PMC8869687

[ref53] Baroi A. M.; Sieniawska E.; Świątek Ł.; Fierascu I. Grape Waste Materials - An Attractive Source for Developing Nanomaterials with Versatile Applications. Nanomaterials. 2023, 13, 836 10.3390/nano13050836. 36903714 PMC10005071

[ref54] Mendes J. A. S.; Xavier A. M. R. B.; Evtuguin D. V.; Lopes L. P. C. Integrated utilization of grape skins from white grape pomaces. Ind. Crops Prod. 2013, 49, 286–291. 10.1016/j.indcrop.2013.05.003.

